# Seamless assembly of recombinant adenoviral genomes from high-copy plasmids

**DOI:** 10.1371/journal.pone.0199563

**Published:** 2018-06-27

**Authors:** Jessica J. Miciak, Jason Hirshberg, Fred Bunz

**Affiliations:** Department of Radiation Oncology and Molecular Radiation Sciences, The Sidney Kimmel Comprehensive Cancer Center, Johns Hopkins University School of Medicine, Baltimore, Maryland, United States of America; Northern University, UNITED STATES

## Abstract

The adenoviruses are essential tools for basic research and therapeutic development. Robust methods for the generation of mutant and recombinant viruses are crucial for these diverse applications. Here we describe a simple plasmid-based method that permits highly efficient modification of the adenoviral genome and rapid production of high-titer virus stocks. The 36-kilobase genome of adenovirus serotype 5 was divided into seven tractable blocks that could be individually modified in a single step and reassembled *in vitro*. Because the system is composed of compact modules, modifications at different loci can be readily recombined. Viral assemblies were delivered to packaging cells by electroporation, a strategy that consistently resulted in the *de novo* production of 10^8^ infectious units in 3–5 days. In principle, a similar strategy could be applied to any other adenovirus serotype or to other double-strand DNA viruses.

## Introduction

Detailed studies of the adenovirus family were foundational for our understanding of eukaryotic DNA replication and gene regulation, and continue to provide important insights into the nature of the host-pathogen interaction [1,2]. These studies also revealed unique attributes that have made adenoviruses suitable for a wide range of translational, clinical and industrial applications. Adenoviruses were among the first human viruses to be harnessed for transgene delivery [3], and have since become indispensable tools for the development of vaccines and novel therapies. The development of oncolytic adenovirus mutants that can exploit the innate immune deficiencies of tumor cells highlights the potential of customized adenoviruses as biological drugs [4].

The adenoviridae comprise a large and relatively diverse family of medium sized, non-enveloped, doubled-stranded DNA viruses, which collectively infect numerous hosts and tissues [1]. More than 50 distinct serotypes have been identified in humans. In immunocompetent individuals adenoviral infections tend to be self-limiting and are often subclinical. Exposure is prevalent throughout the human population, and most adults have acquired immunity to multiple adenovirus serotypes. Among the most frequently studied and heavily exploited of the human adenoviruses is serotype 5 (Ad5). The 35,938 bp genome of Ad5 encodes 36 proteins.

Ad5 and related serotypes can be reconfigured to deliver large transgenes with high efficiency. Once unpackaged inside the cell, the adenoviral genome is maintained transiently as an episome, and does not integrate into the genome of the host. Recombinant adenoviruses designed to deliver transgenes commonly lack some of the mechanisms that wild-type adenoviruses employ to circumvent host immunity and are most often replication-incompetent. Together, these attributes make recombinant adenoviruses a popular vector for transgene delivery.

Traditionally, the production of adenoviral vectors was a laborious process involving homologous integration of mutant sequences or transgenes in human packaging cells followed by plaque purification and screening for clonal recombinants. A major advance was the development of cloned viral DNAs that could be propagated in bacteria or yeast and then transferred to specialized human cells for packaging [5–8]. Subsequently, an *in vivo* approach known as recombineering permitted desired genetic elements to be seamlessly incorporated into viral genomes via homologous recombination in bacteria. A number of recombineering systems have been introduced that employ recombogenic bacterial strains and specialized features such as lambda phage-mediated gene transfer and strategies for vector excision [9–12]. Such systems have facilitated the development of high-capacity adenoviral vectors (so-called “gutless” vectors), which typically employ a shuttling system for large transgenes and a helper virus that provides essential viral proteins in *trans* [12–14]. Recently, CRISPR-Cas9 has been used to stimulate site-specific recombination directly in packaging cells [15]. The genomic modifications enabled by these increasingly powerful methods of recombineering have been extensively used to modify viral characteristics and improve our understanding of viral gene function [16–18].

Enzymatic methods have also been employed for viral genome assembly. A distinct advantage of *in vitro* assembly is that synthetic DNAs can be directly incorporated into the viral genome. Infectious adenoviruses have been rescued from genomes that were assembled *in vitro* from multiple cloned segments, initially derived by high-fidelity PCR [19]. A synthetic approach is particularly well-suited for the PCR amplification and reassembly of viruses isolated from clinical samples. Mutations or transgenes can be simultaneously introduced into multiple sites across a viral genome, and DNA sequencing can be easily performed on subcloned viral segments prior to their assembly.

**Fig 1:**
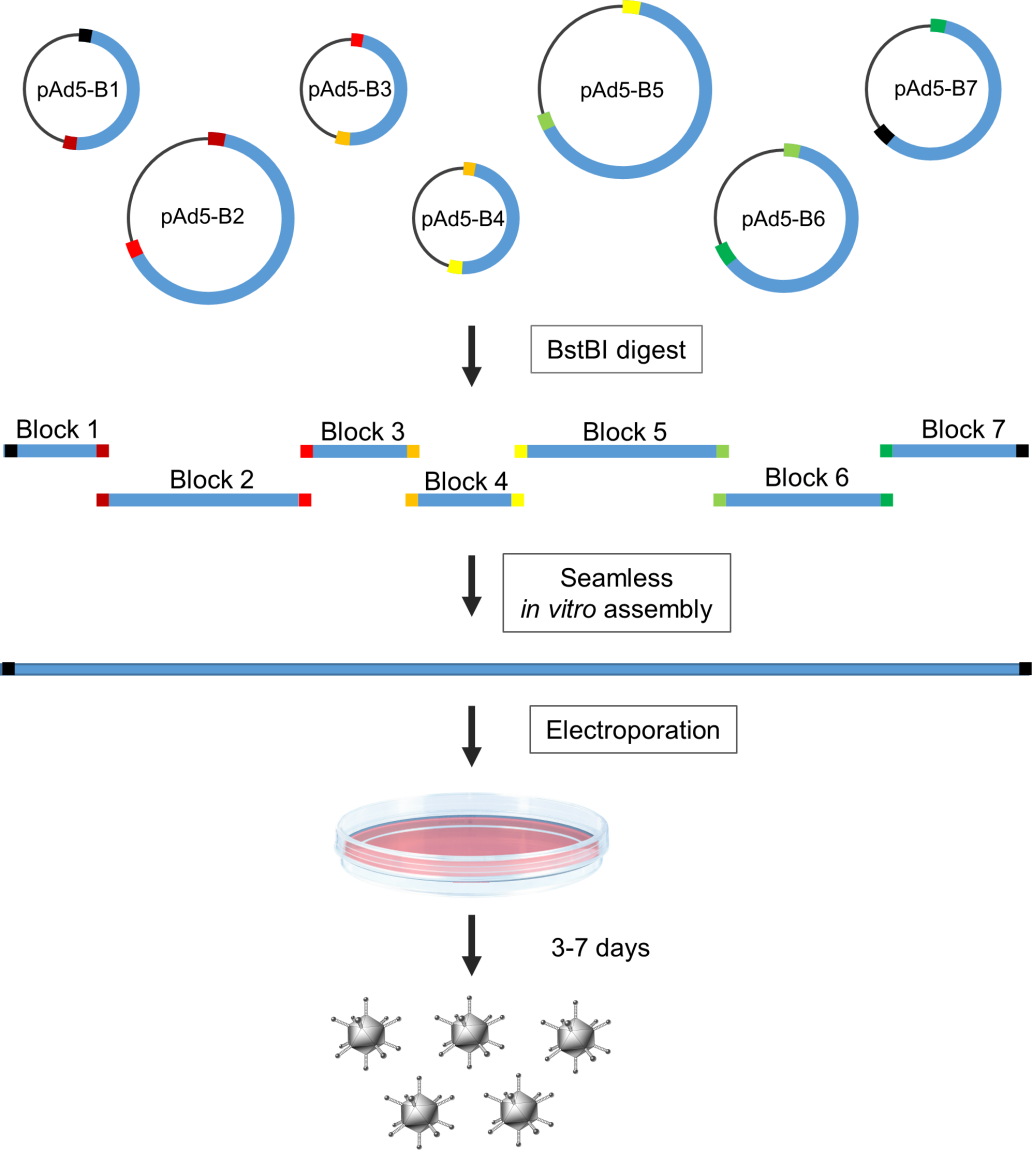
Overview of the Adenobuilder system. Segments of the Ad5 genome were individually amplified and cloned into high-copy plasmids. Each of the five central “building blocks” contains unique sequences at either end (represented by distinct colors) that create a 15–20 bp overlap with the respective neighboring block. Each block is flanked by recognition sites for the restriction enzyme BstBI. Following digestion, the unpurified plasmid fragments are directly added to an isothermal assembly reaction and then delivered to packaging cells via electroporation. This system thus facilitates the generation of infectious adenovirus particles from plasmids in three steps that can be carried out in under two hours.

Here, we describe a synthetic system for viral genome assembly and packaging that we call Adenobuilder. This system permits the rapid generation of high-titer Ad5 stocks from ordinary high-copy plasmids (Fig 1). The partitioning of the Ad5 genome into seven modular “blocks” allows individual viral genes to be easily manipulated in isolation and then recombined with other mutations or transgenes. When delivered to complementary packaging cells by electroporation, the enzymatically assembled genomes were packaged at high efficiency. Unlike recombineering systems, the Adenobuilder method does not require specialized bacterial strains or the propagation of low copy vectors such as bacterial artificial chromosomes.

## Materials and methods

### Ad5 virus, cell lines, and antibodies

Ad5 reference virus (catalog VR-1516) and HEK293 cells (catalog CRL-1573) were obtained directly from the American Type Tissue Collection. W162 cells were a gift from Gary Ketner, who originally derived them from the cell line Vero [20]. Both cell lines were maintained at 37°C under 5% CO_2_ in Dulbecco’s Modified Eagle Medium with high glucose and pyruvate, supplemented with 10% fetal bovine serum and penicillin/streptomycin. A puromycin-sensitive subclone of the cell line hTERT-RPE1 was a gift from Andrew Holland, and was phenotypically validated. The hTERT-RPE1 cells were cultured in DMEM/F-12. Culture media and all additives were purchased from Thermo Fisher Scientific. The mouse monoclonal antibody DO-1 used for detection of p53 was purchased from Santa Cruz Biotechnology (catalog sc-126). An antibody that recognizes the FLAG epitope was purchased from Cell Signaling Technologies (catalog 14793). Immunofluorescence was performed with a FITC-conjugated polyclonal antibody directed against Ad5 hexon purchased from Thermo Fisher Scientific (catalog PA1-73053). The polyclonal antiserum against the Ad5 E4ORF3 protein was a gift from Gary Ketner.

### Amplification of Ad5 genomic DNA segments

HEK293 cells were infected with wild type Ad5 at a multiplicity of infection of 10, incubated for 48 h and then harvested with 0.05% trypsin-EDTA. Genomic DNA was purified over a spin column (Thermo Fisher Scientific) and used as a PCR template at a final concentration of 4 ng/μl. Overlapping regions of Ad5 genomic DNA were amplified with Platinum SuperFi DNA polymerase (Thermo Fisher Scientific). PCR primer sequences and conditions for the synthesis of each block are listed in S1, S2 and S3 Tables.

### Plasmid construction

Gel-purified PCR products were directly ligated into the blunt-ended pJET1.2 cloning vector (Thermo Fisher Scientific) as per the manufacturer’s protocols. The resulting plasmids were purified by a mini-column procedure, assessed by restriction mapping and verified by Sanger sequencing. The primers used for insert sequencing are listed on S4 Table. Complete plasmid sequences and mapping information are provided in the S1 Fig.

Plasmids with mutant viral sequences were generated by PCR, using the corresponding wild type blocks as templates. Amplification was performed with Phusion, an engineered high-fidelity DNA polymerase with enhanced processivity, using the buffers and additives supplied by the manufacturer (New England Biolabs). Each 20 μl reaction contained 200 μM of each dNTP, 0.5 μM of each primer, 0.2 U of enzyme and 1 ng supercoiled plasmid DNA template. After heating at 98°C for 1 min, reactions were subjected to a total of 20 cycles of amplification. The first five cycles included a 5 s denaturation step at 98°C, a 10 s annealing step at 62°C, and a 4 min step for extension at 72°C. The annealing temperature was increased to 68°C for the remaining 15 cycles, which were otherwise identical. Input plasmid DNA templates were digested with 20 U of the restriction enzyme DpnI (New England Biolabs) for 30 min at 37°C. PCR products of the expected sizes were gel purified and eluted from a spin column in 30 μl of water. Circularization of the purified PCR products was performed with the NEBuilder HiFi DNA Assembly Cloning kit (New England Biolabs). The 20 μl reaction, containing 10 μl PCR DNA (5–20 ng) and 10 μl 2X NEBuilder HiFi Assembly Master Mix, was incubated at 50°C for 10 min. Following assembly, 0.5 μl of the reaction was directly delivered to competent DH10β cells (New England Biolabs) by electroporation. Isolated plasmids were verified by Sanger sequencing.

### Assembly of Ad5 genomes

Plasmid concentrations were determined with a spectrophotometer (NanoDrop, Thermo Fisher Scientific). A DNA mixture containing 125 fmol of each of the seven plasmids (4.4 μg DNA in total) was digested in a 50 μl reaction containing 50 U of the restriction enzyme BstBI (New England Biolabs). The digest was incubated at 50°C for 30 min. Next, 50 μl of 2X NEBuilder HiFi Assembly Master Mix was added. The reaction was briefly mixed and then returned to 50°C for 1 h. Following assembly, all DNAs were precipitated in 0.5 M ammonium acetate and 70% ethanol. Pelleted DNA was washed with 70% ethanol and stored at -20°C prior to electroporation.

For analysis of assembly efficiency, 8.8 μg of pooled plasmid DNA from each of the seven blocks was digested with BstBI. After 30 min at 50°C, one half of the digest was transferred to a second tube and combined with the enzyme components required for assembly. Both tubes were incubated at 50°C for an additional 60 min and then put on ice. An equal proportion of each mixture was analyzed on a 0.8% agarose gel run at 40 V for 11 h. Mono cut bacteriophage lambda DNA and 1 kb-plus ladder (New England Biolabs) were run as size markers.

### Electroporation of packaging cells

DNAs were delivered to HEK293 cells by electroporation with a 4D-Nucleofector apparatus (Lonza) as per the manufacturer’s recommendations. Cells were seeded in standard culture flasks 48 h prior to electroporation and harvested by incubation with trypsin-EDTA when 80–90% confluent. Approximately 2.0E+6 cells were added to complete medium and pelleted at 90 g for 10 minutes at room temperature. DNA pellets were thawed, dissolved in 10 μl of sterile water and brought to 100 μl in freshly supplemented Nucleofector solution SF (Lonza). Each cell pellet was gently resuspended in the DNA solution and transferred to a Nanocuvette (Lonza). The mixture of cells and DNA was subjected to the preprogrammed pulse CM130. Cells were transferred to pre-warmed medium in a T25 flask immediately following electroporation.

### Ad5 preparation, titration and analysis

Flasks were scraped 3–5 days after delivery of the assembled DNAs, when the packaging cells appeared to be detaching and the medium was fully yellow. Detached cells and culture media were removed together and immediately frozen in a dry ice/ethanol bath. This lysate was thawed, vortexed for 15 s and frozen two additional times. Following the last thaw, the sample was clarified by centrifugation in a HS-4 rotor (Sorvall) run at 7000 RPM (10826 g) for 10 min at 4°C. The virus-containing supernatant was removed and stored at -80°C.

For the assessment of viral titer, packaging cells were seeded a standard 24-well cell culture plate (Corning Costar) 24–48 h prior to infection and grown to near-confluence. Cells were infected with serially diluted lysate. After incubation for 24 h, the cell monolayer was washed once in PBS, and then simultaneously fixed and permeabilized in a PBS solution containing 3% paraformaldehyde and 0.1% Triton-X100 for 30 min at room temperature. Following fixation and permeabilization, cells were washed two times with PBS and incubated with a FITC-conjugated antibody against the Ad5 hexon protein, which was diluted 1:100 in a PBS solution containing 2% bovine serum albumin. Stained cells were visualized and counted under fluorescence with an EVOS Cell Imaging System (Thermo Fisher Scientific).

Adenoviral genomes were mapped by restriction digests containing 20 U enzyme and 1 μg DNA, purified from pelleted viral particles as described [21]. For assessment by electron microscopy (EM), virus-containing lysate was directly applied to a grid and negatively stained with 1% phosphotungstic acid.

## Results and discussion

The technique developed by Gibson and colleagues [22] for the seamless assembly of overlapping linear DNAs has found many applications in the field of synthetic biology. Here, we demonstrate how slight refinements of this technique can greatly simplify the creation of adenoviral mutants and the subsequent modular assembly and packaging of viral genomes.

**Fig 2:**
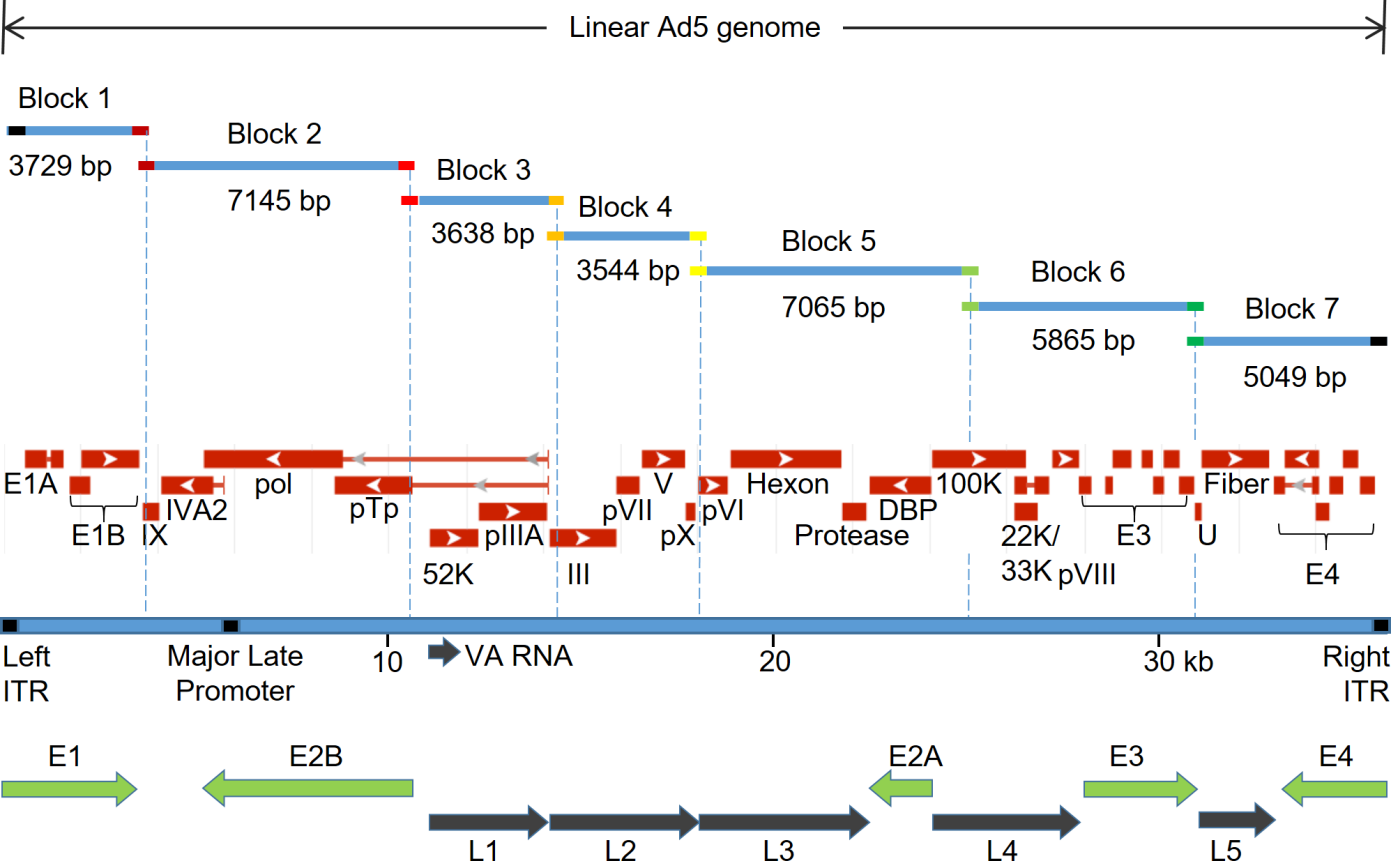
Partitioning of the Ad5 genome. The cloned blocks 1–7 are aligned to a map of the Ad5 genome. Protein-coding genes are indicated in red, with arrows indicating the strand that is transcribed. The inverted terminal repeats (ITRs), which define the left and right ends of the adenovirus episome, and virus-associated RNAs (VA RNA) are shown. The early and late transcriptional units are marked by green and black arrows, respectively.

In the design of the Adenobuilder system, we chose to partition the adenoviral genome into blocks that each contain a defined transcription unit (Fig 2). The sizes of the blocks ranged from 3.5 to 7.1 kb, and thus were small enough to be robustly amplified by high-fidelity PCR and stably cloned into plasmids that could be propagated at high copy number. The compiled sequence of the seven cloned blocks differed from the Ad5 reference sequence (NCBI AC_000008.1) at 19 positions (S4 and S5 Tables). Further analysis of independent clones and direct sequencing of PCR products revealed that 18 of these putative mutations were present in the original, unamplified virus that was obtained from ATCC (S5 Table). The remarkably high fidelity of PCR amplification was consistent with error rates reported by the enzyme manufacturer.

Previous approaches to the synthesis of adenovirus genomes from cloned DNAs have used 2–4 blocks that were necessarily larger in size, presumably to facilitate efficient assembly. However, this presumed requirement for efficiency comes at the price of working with large DNA blocks that are significantly more difficult to modify and stably propagate.

**Fig 3:**
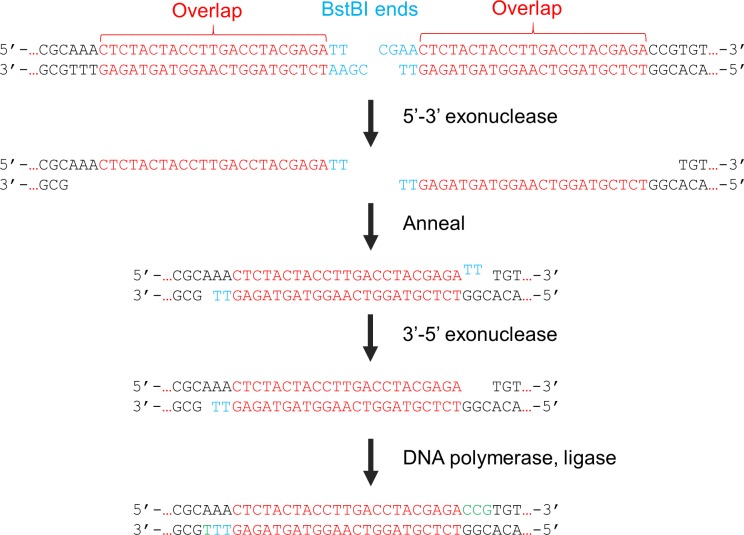
Seamless ligation of adjacent genomic blocks. The free ends created by BstBI are initially processed by the heat-labile T5 exonuclease, creating an extended 3’ overhang at each end. When complementary strands anneal, the ends are trimmed, filled in and ligated. The thermostable BstBI enzyme remains active during the assembly process and thus prevents re-ligation of the cut ends. The compatible ends of Blocks 1 and 2 are shown; the other junctions are simultaneously processed in the same manner.

Each cloned block was flanked with the recognition sequence for the restriction enzyme BstBI, which is not present in the Ad5 reference genome. This enzyme was originally derived from the thermophile *Bacillus stearothermophilus* and is optimally active at elevated temperatures. This property facilitates a simple isothermal assembly process. Following BstBI digestion, assembly was performed by a combination of enzymes that includes a mesophilic 5’-3’ exonuclease, a high-fidelity thermophilic DNA polymerase with 3’-5’ exonuclease activity and a thermophilic DNA ligase. Similar to the approach first devised by Gibson [22], these enzymes coordinately create complementary single-stranded regions at overlapping termini, remove small mismatches at the ends once the strands anneal, fill in the gaps and ligate the ends (Fig 3). These reactions are performed at 50°C. Because the 5’-3’ exonuclease is labile at the reaction temperature, the generation of single stranded regions is transient. The remaining enzymes, including BstBI, remain active and promote assembly to completion.

**Fig 4:**
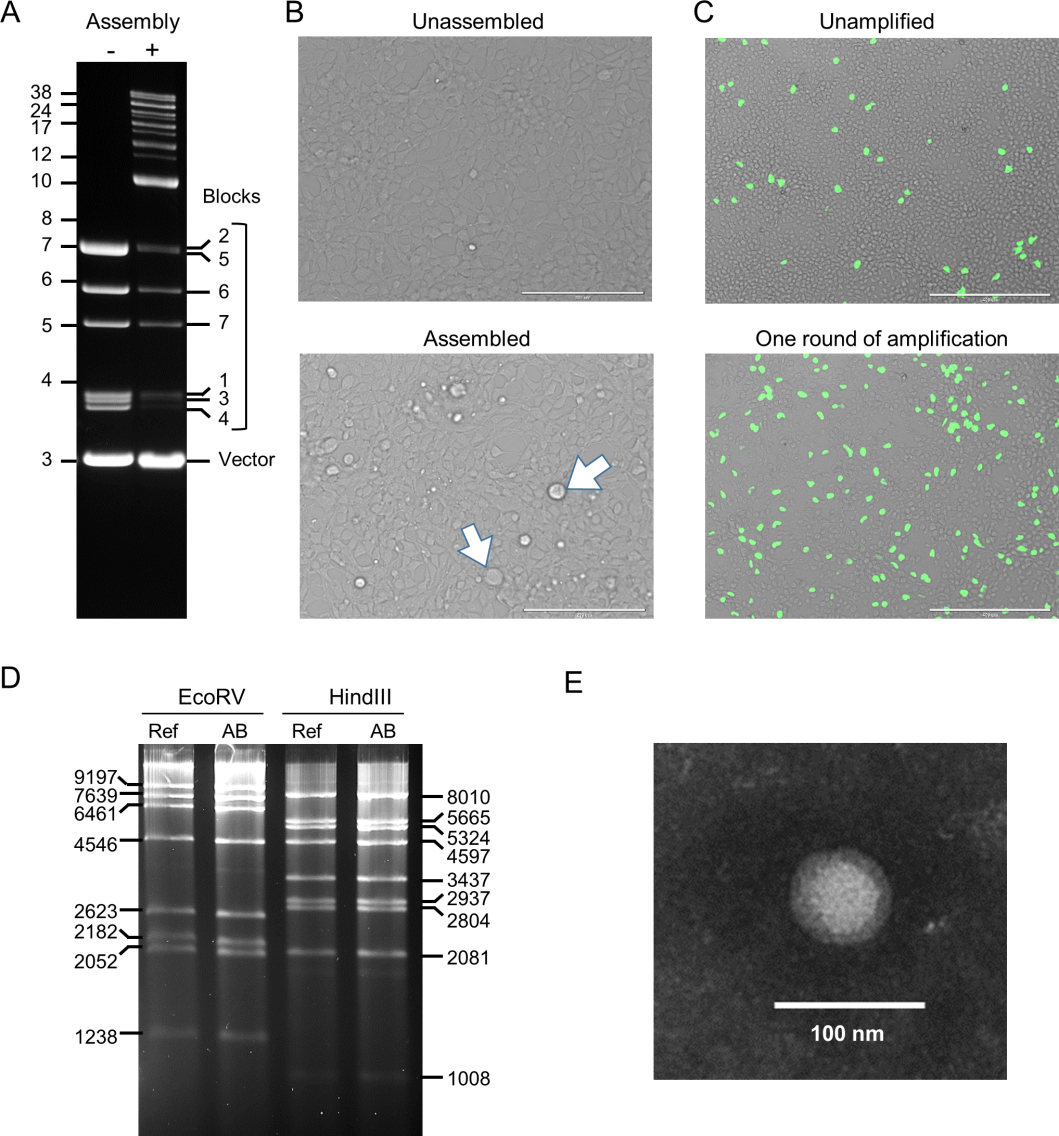
Reassembly and packaging of infectious Ad5. **A.** Equimolar amounts of each of the seven Adenobuilder plasmids were digested with BstBI and then incubated for an additional 60 min with (+) and without (-) the assembly enzyme mix. Aliquots containing 660 ng total DNA were assessed on a 0.8% agarose gel. Size markers (in kb) are shown on the left; the bands corresponding to the vector backbone and each of the excised blocks is indicated at right. **B.** Unassembled (upper panel) or assembled (lower panel) blocks were delivered to HEK293 cells by electroporation. Images were captured at 36 h, at 20X magnification. Arrows indicate representative rounded cells. Scale bar = 200 μm. **C.** HEK293 cells were infected with 10 μl of the primary lysate, derived directly from cells that had been electroporated (upper panel), or with 1 μl of lysate obtained after a single round of amplification (lower panel). Viral titers were determined by detection of the Ad5 hexon protein (green), which is expressed late in the viral life cycle. Magnification, 10X. Scale bar = 400 μm. **D.** Genomic DNAs from the reference Ad5 stock (Ref) and assembled Ad5 (AB) were compared by restriction digest. One μg of DNA from each sample was digested with EcoRV or HindIII. Fragments of the indicated sizes were resolved on 0.8% agarose. **E.** A viral particle in a primary, unpurified lysate was visualized by electron microscopy after negative staining, Magnification, 135000X. Scale bar = 100 nm.

We first generated infectious virus from unmodified blocks that were derived directly from the wild type reference Ad5 genome. As described in Materials and methods, the seven plasmids were combined in a single BstBI digestion reaction and incubated at 50°C. All plasmids appeared to be completely cut after 30 min, and each excised block could be readily visualized on an agarose gel (Fig 4A). The assembly reaction generated a ladder of high molecular weight DNAs, with the uppermost band running at the approximate size of the full-length Ad5 genome. The intermediate bands between the unit blocks and the full length genome presumably reflected partial assemblies. Pairwise assembly of Blocks 1–2, 2–3, 4–5, and 6–7 would account for the prominent band running at about 10 kb (Fig 4A). Overall, the products of the assembly reaction were biased in favor of partially assembled DNAs. However, because the input DNA blocks are present at picomolar levels, significant amounts of full length DNA could be generated. Adenoviruses only package genomes within a limited size range. Presumably any partial assembly would be lacking critical functional elements required for productive infection and replication.

A cytopathic effect was evident 2–3 days after electroporation in cells that were transfected with assembled DNA (Fig 4B). Between 4–7 days post infection, many cells became detached and the medium appeared acidic, observations that are consistent with active virus production. Analysis of these primary cultures indicated that viruses were generated in amounts ranging between 10^6^−10^7^ IU. A 200 μl aliquot of a primary Ad5 lysate, about 5% of the total lysate obtained, was applied to a single flask containing 2 x 10^6^ HEK293 cells. These cells were harvested after 72 h. Viral titers increased exponentially, to 10^8^−10^9^ IU, after this single small-scale round of amplification (Fig 4C), indicating that the newly packaged viruses were functional. To confirm that the blocks had assembled as expected, we compared viral genomic DNAs from the original Ad5 reference stock (ATCC) and the amplified stock of assembled virus by restriction digest. Incubation with EcoRV and HindIII generated all of the expected bands in both samples (Fig 4D). (A 75 bp band expected from the HindIII digest could not be resolved on this type of gel.) Individual viral particles of the expected size could be detected in the primary lysate by EM (Fig 4E). For various assemblies, we found that a stock with a useable titer could be reliably generated within a week of electroporation.

**Fig 5:**
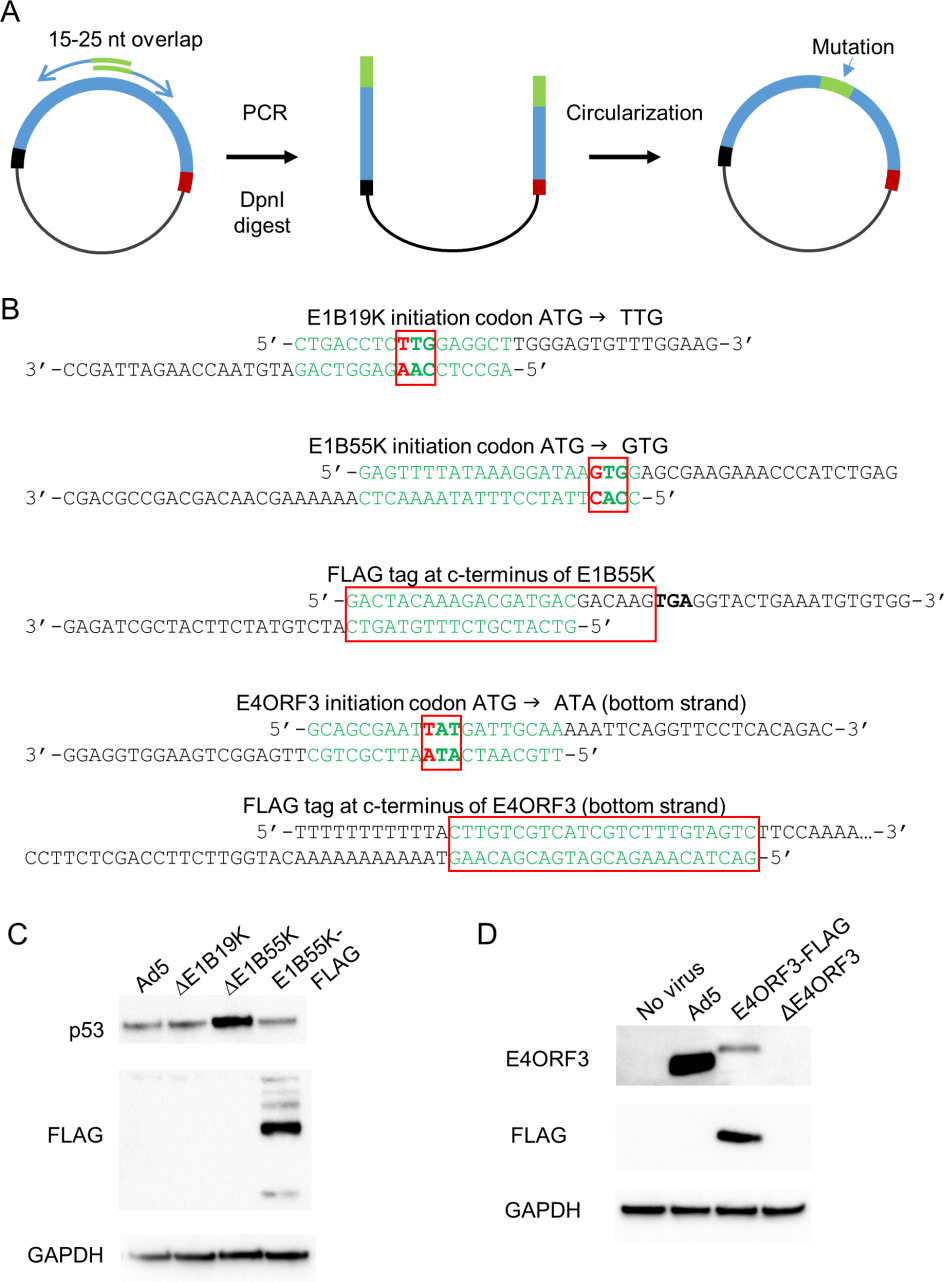
Two-step derivation of mutations in E1B and E4. **A.** The mutant constructs were first amplified with two partially overlapping primers designed to incorporate the desired alteration (shown in green), using the corresponding wild type block as a template. The input template DNA was then eliminated by digestion with the restriction enzyme DpnI. In the second step, the PCR-derived plasmid was circularized in an assembly reaction, which merged and sealed the overlapping ends. **B.** Oligonucleotide pairs that were used to generate the mutants are illustrated. The mutant block 1-derived constructs were E1B19K-null, E1B55K-null or modified to express an endogenous E1B55K protein with a c-terminal FLAG epitope. Similarly, the E4ORF3 open reading frame was disrupted by a mutation at the initiation codon or the introduction of a c-terminal FLAG tag. The targeted codons are boxed, single base mutations are shown in red. At positions where the initiation codon of the target protein overlapped with a codon for a different protein, base substitutions were selected so that the mutation would be silent with respect to the second open reading frame. **C.** hTERT-RPE1 cells were infected with the synthetic Ad5 virus and the E1B mutant viruses generated in this study. Cells were lysed 16 h post-infection, and assessed by immunoblot with antibodies against p53 and the FLAG epitope, as indicated. **D.** hTERT-RPE1 cells were uninfected (no virus) or infected with wild type Ad5 and the E4 mutants. For all viruses, the MOI was 100. GAPDH was probed as a loading control.

The relatively small size of the blocks makes them easy to modify. To demonstrate a straightforward approach to mutagenesis (Fig 5A), we incorporated several functional base changes into the E1B and E4 loci, respectively located on Blocks1 and 7 (Fig 5B). The Gibson method is often used to assemble plasmids from multiple overlapping fragments, but can also be employed to circularize plasmids generated by PCR. We exploited this attribute, using overlapping oligonucleotides to incorporate mutations into derivative plasmids. Each oligonucleotide pair was designed with altered bases in an overlapping region such that high-fidelity PCR amplification from a plasmid template resulted in a linear DNA with a mutation of interest reproduced at both ends. The linear DNAs were then circularized in an isothermal reaction that was similar to that used for multi-block genome assembly. This method proved to be remarkably efficient. In our hands, nearly 100% of the plasmid subclones harbored the desired alteration.

The E1 locus encodes several oncogenic proteins that are crucial for bypassing the innate immune responses of the infected host cell. The E1B55K protein, in cooperation with E4ORF6, recruits several host proteins to form an active ubiquitin ligase complex that binds and degrades p53 protein [23]. Adenoviruses with mutant E1B55K fail to suppress the activation of p53, and therefore cause a marked increase in p53 abundance. Following assembly, packaging and a single round of amplification, the E1B-mutant viruses were used to infect hTERT-RPE1, an immortalized human cell line derived from retinal epithelia. These cells are not cancer-derived, and express functional p53 [24]. Infection with the synthetically-derived mutant E1B viruses demonstrated the known requirement for the E1B55K protein, but not the E1B19K protein, for the inhibition of p53 (Fig 5C). Interestingly, the endogenous c-terminal FLAG tag did not significantly impair the ability of the E1B55K to participate in the degradation of p53.

Like E1, the early region E4 is critical for inactivation of the p53 pathway in infected cells. One of the small proteins expressed from the E4 locus, E4ORF3 forms a nuclear scaffold that causes the epigenetic silencing of p53 target genes [25]. The generation of mutations within E4 presents a distinct challenge, as HEK293 cells do not normally complement losses of function within this region. In order to derive E4ORF3-mutant viruses, we first attempted to deliver assembled viral genomes directly into the E4-complementing cell line W162 [20]. These experiments did not yield infectious virus. Next, we mixed the E4-mutant assemblies with 1 μg of the undigested pAd5-B7 plasmid, which harbors the wild type E4 region, just prior to electroporation of HEK293. This *trans*-complementation approach resulted in the production of infectious virus with alterations in E4. We successfully produced a mutant that was null for E4ORF3 as well as a virus that expressed a FLAG-tagged E4ORF3 protein from its endogenous position in E4 (S2 Fig). The E4ORF3-FLAG protein could be readily detected following infection of hTERT-RPE1, albeit at lower levels than the wild type, untagged E4ORF3 protein (Fig 5D). Once packaged in HEK293, these viruses could be robustly amplified in W162 cells, allowing the production of high-titer stocks. While most of the lysates contained viruses that exclusively harbored the desired E4 mutations, wild type sequences were sporadically detected when this entire assembly-packaging process was repeated numerous times, indicating that mutant assemblies recombined with the input wild type plasmid at a detectable frequency.

**Fig 6:**
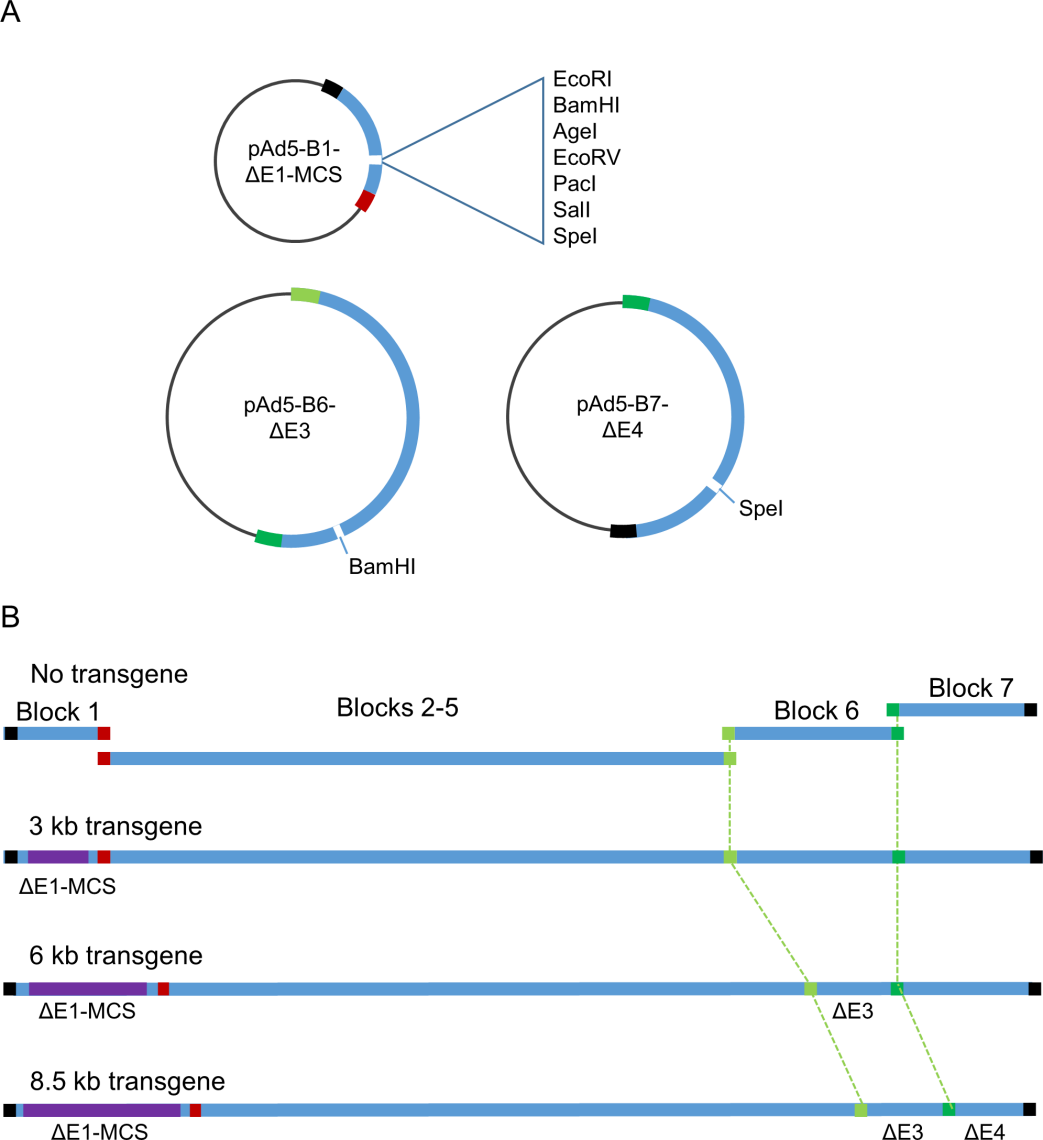
Modifications to blocks 1, 6 and 7 facilitate the incorporation of large transgenic elements into recombinant Ad5 vectors. **A**, DNA cassettes up to 3 kb in size can be directly cloned into a polylinker in the modified block pAd5-B1ΔE1-MCS. Still larger transgenic DNAs can be incorporated into assembled viruses with the use of plasmids pAd5-B6ΔE3 and/or pAd5-B7ΔE4. **B**, successively larger transgenes can be introduced with the deletion of E1, E3 and E4. Used together, these three modified blocks thus allow the customized assembly of recombinant viruses capable of delivering transgenic elements up to 8.5 kb in size.

Because adenoviral vectors are most frequently employed for transgene delivery, we modified the wild type Adenobuilder blocks so that they could accommodate exogenous DNA cassettes of various sizes (Fig 6A). Expression cassettes up to 3 kb can be directly inserted pAd5-B1ΔE1-MCS, which contains a convenient polylinker that replaces a similarly-sized region in E1. The potential delivery capacity of Adenobuilder-derived vectors was further increased by deleting endogenous loci in blocks E3 and E4. Incorporating pAd5-B6ΔE3 and pAd5-B7ΔE4 into a synthetic assembly permits the packaging of synthetic adenoviruses that harbor transgenic elements up to 8.5 kb in size, as depicted in Fig 6B. The only limitation other than size would be the requirement that inserted cassettes be designed so as not to contain the recognition site for BstBI.

While numerous techniques for the development of customized adenoviruses are currently available, the Adenobuilder method has several advantages. Because the adenoviral genome is partitioned into 7 compact, functionally-defined modules, each individual module is small enough to be stably propagated on a conventional, high-copy plasmid. Such plasmids are easily prepared, stored and modified by procedures that are standardized and highly accessible. We found that virus production following synthetic genome delivery was highly robust, which allowed us to minimize the iterative rounds of amplification that are commonly needed to produce stocks of useable titer.

In these studies we introduced various mutations, epitope tags and polylinker elements into the blocks corresponding to E1, E3 and E4. The trans-complementation approach successfully used to generate E4 mutants could, in principle, be applied to the creation of mutations in other Ad5 segments. Such mutants were not generated in this course of the present study. The region between E1 and E4 encompasses more than one half of the viral genome (Fig 1) and harbors many genes and promoter elements that are essential for viral propagation. Despite decades of intense investigation, the functions of numerous adenovirus genes and proteins remain poorly understood. The ability to quickly and easily generate mutants in these regions could accelerate the pace of research and hasten the development new and improved adenoviral vectors.

## Supporting information

S1 Table PCR primers and conditions for segmental amplification of the Ad5 reference genome. (DOCX)

S2 Table PCR primers used to amplify mutant blocks. (DOCX)

S3 Table Sequencing primers used to confirm mutations. (DOCX)

S4 Table Primers for sequencing plasmid inserts. (DOCX)

S5 Table Sequence variance from NCBI Reference Sequence AC_000008.1. (DOCX)

S6 Table Primers used to amplify E4 region from the packaged viral genome. (DOCX)

S1 Fig Annotated plasmid DNA sequences. (DOCX)

S2 Fig Sequencing E4 mutant viral DNAs. (DOCX)
