# Supplementary material for: Seamless assembly of recombinant adenoviral genomes from high-copy plasmids
Source: PLoS One. 2018 Jun 27;13(6):e0199563. doi: 10.1371/journal.pone.0199563 (PMC6021080; doi:10.1371/journal.pone.0199563)
Supplement: S1 Table — (DOCX) [file pone.0199563.s001.docx]

**S1 Table**

**PCR primers and conditions for segmental amplification of the Ad5 reference genome**

The recognition sequence for BstBI in each primer is highlighted. The site highlighted in red is the 5’ end of the amplicon, with respect to the Ad5 reference sequence AC_00008.1.

PCR was performed with Platinum SuperFi DNA polymerase (Thermo Fisher Scientific) using the supplied buffers and additives according to the manufacturer’s recommendations. Each 25 μl reaction contained 0.2 μM of each dNTP, 0.5 μM of each primer, 0.2 U of enzyme and 100 ng Ad5 genomic DNA template. After heating at 95 C for 2 min, reactions were subjected to 35 cycles of amplification. Each cycle consisted of a 10 s denaturation step at 95 C, and annealing for 10 s. Annealing temperatures and extension times per cycle are indicated in the table below.

| Block | Primer | Sequence | Amplicon size (bp) | Annealing Temp (°C) | Extension time (min:sec) |
| --- | --- | --- | --- | --- | --- |
| 1 | **AB-1** | cttcgaaCATCATCAATAATATACCTTATTTTGG | 3773 | 58 | 2:45 |
|  | **AB-2** | gttcgaaTCTCGTAGGTCAAGGTAGTA |  |  |  |
| 2 | **AB-3** | cttcgaaCTCTACTACCTTGACCTACG | 7159 | 58 | 4:40 |
|  | **AB-4** | gttcgaaCGAGCCACTTAATGCTTTC |  |  |  |
| 3 | **AB-5** | cttcgaaCGAAAGCATTAAGTGGCTC | 3652 | 58 | 2:45 |
|  | **AB-6** | gttcgaaTCGTCAAGATTGATGGTCTGT |  |  |  |
| 4 | **AB-7** | cttcgaaGCACACAGACCATCAATCT | 3557 | 60 | 2:45 |
|  | **AB-8** | gttcgaAGTTGATGTCTTCCATTCTAC |  |  |  |
| 5 | **AB-9** | cttcgaaGTAGAATGGAAGACATCAACT | 7079 | 58 | 4:40 |
|  | **AB-10** | gttcgaaCATCATTAGTTTGCGTCGCT |  |  |  |
| 6 | **AB-11** | cttcgaAGCGACGCAAACTAATGATG | 5878 | 58 | 3:40 |
|  | **AB-12** | gttcgaaTGCTGAATAAACTGGACAG |  |  |  |
| 7 | **AB-13** | cttcgaaCTGTCCAGTTTATTCAGCA | 5064 | 58 | 3:40 |
|  | **AB-14** | gttcgaaCATCATCAATAATATACCTTATTTTGG |  |  |  |
