# Supplementary material for: Seamless assembly of recombinant adenoviral genomes from high-copy plasmids
Source: PLoS One. 2018 Jun 27;13(6):e0199563. doi: 10.1371/journal.pone.0199563 (PMC6021080; doi:10.1371/journal.pone.0199563)
Supplement: S2 Table — (DOCX) [file pone.0199563.s002.docx]

**S2 Table**

**PCR primers used to amplify mutant blocks**

The inserted/mutated sequences are highlighted in green. The overlap between each primer pair is underlined.

| Gene | Block | Alteration | Primer | Sequence |
| --- | --- | --- | --- | --- |
| E1B55K | 1 | Insert c-terminal FLAG tag | **Ad5-10** | GACTACAAAGACGATGACGACAAGTGAGGTACTGAAATGTGTGG |
|  |  |  | **Ad5-11** | GTCATCGTCTTTGTAGTCATCTGTATCTTCATCGCTAGAG |
| E1B19K | 1 | ATG 🡒 TTG at first codon | **Ad5-12** | CTGACCTCTTGGAGGCTTGGGAGTGTTTGGAAG |
|  |  |  | **Ad5-13** | AGCCTCCAAGAGGTCAGATGTAACCAAGATTAGCC |
| E1B55K | 1 | ATG 🡒 GTG at first codon | **Ad5-14** | GAGTTTTATAAAGGATAAGTGGAGCGAAGAAACCCATCTGAG |
|  |  |  | **Ad5-15** | CCACTTATCCTTTATAAAACTCAAAAAAGCAACAGCAGCCGCAGC |
| E4ORF3 | 4 | ATG 🡒 ATT at first codon | **Ad5-19** | GCAGCGAATTATGATTGCAAAAATTCAGGTTCCTCACAGAC |
|  |  |  | **Ad5-20** | TTGCAATCATAATTCGCTGCTTGAGGCTGAAGGTGGAGG |
| E4ORF3-FLAG | 4 | Insert c-terminal FLAG tag | **Ad5-26** | TTTTTTTTTTTACTTGTCGTCATCGTCTTTGTAGTCTTCCAAAAGATTATCCAAAACCTC |
|  |  |  | **Ad5-27** | GACTACAAAGACGATGACGACAAGTAAAAAAAAAAACATGGTTCTTCCAGCTCTTCC |
