# Supplementary material for: Seamless assembly of recombinant adenoviral genomes from high-copy plasmids
Source: PLoS One. 2018 Jun 27;13(6):e0199563. doi: 10.1371/journal.pone.0199563 (PMC6021080; doi:10.1371/journal.pone.0199563)
Supplement: S3 Table — (DOCX) [file pone.0199563.s003.docx]

**S3 Table**

**Sequencing primers to confirm engineered mutations**

| Block | Gene | Primer | Sequence |
| --- | --- | --- | --- |
| 1 | **E1B19K** | **Ad5-16** | CTTGCATGGCGTGTTAAATGG |
| 1 | **E1B55K**  **n-terminus** | **Ad5-17** | CAAGAGAAGGTCATCAAGAC |
| 1 | **E1B55K**  **c-terminus** | **Ad5-18** | GGATGTGACCGAGGAGCTGAG |
| 7 | **E4ORF6**  **E4ORF3** | **Ad5-23** | TACGGAGTGCGCCGAGACAAC |
| 7 | **E4ORF3** | **Ad5-24** | TCTCATCTCGCCACCTTCTC |
