# Supplementary material for: Seamless assembly of recombinant adenoviral genomes from high-copy plasmids
Source: PLoS One. 2018 Jun 27;13(6):e0199563. doi: 10.1371/journal.pone.0199563 (PMC6021080; doi:10.1371/journal.pone.0199563)
Supplement: S4 Table — (DOCX) [file pone.0199563.s004.docx]

**S4 Table**

**Primers for sequencing plasmid inserts**

|  | **Primer** | **Sequence** |
| --- | --- | --- |
| Block 1 | B1_1 | GAGGGTGAGGAGTTTGTGTTAG |
|  | B1_2 | CTACAGAGGAGGCTAGGAATCT |
|  | B1_3 | CTGCCACGGAGGTGTTATTA |
|  | B1_4 | GGTATCCTGTCTGAGGGTAACT |
|  | B1_5 | CTCATCCCAGGCAAAGTTAGT |
|  | B1_6 | GGTCAGCTGCTCTATGGAATAC |
|  | B1_7 | ATGCCAAACCTTGTACCGGA |
|  | B1_8 | CAGCAGGAGGAAGCCAGG |
| Block 2 | B2_1 | GACAAGCAGCCGAAGTAGAA |
|  | B2_2 | GCCCTTATGGAAGAGGAGATTG |
|  | B2_3 | GAACTCAAAGCGTGGGAAATG |
|  | B2_4 | TTCCAGCGTCCAACCATATC |
|  | B2_5 | TTGCGACTGTGACTGGTTAG |
|  | B2_6 | CAGAGTGGTCCGAGTTTCTATAC |
|  | B2_7 | CGTATTTGACTCGAGGGCTTAC |
|  | B2_8 | GCTCACCTACGAGGAACTTAAA |
|  | B2_9 | CGTTGAGGGTCCTGTGTATTT |
|  | B2_10 | TTTGGCTGGTGGTCTTCTAC |
|  | B2_11 | TGCCAGACTGCGGTATAATG |
|  | B2_12 | CATAGTCCAGCCCCTCCG |
|  | B2_13 | CTCGTATGGGTTGAGTGGGG |
|  | B2_14 | CACGAGCTGCTTCCCAAA |
|  | B2_15 | GCTAACGATGCATCTCAACAAT |
|  | B2_16 | CCTTTTGCACGGTCTAGAGC |
| Block 3 | B3_1 | GGAATATGACGAGGACGATGAG |
|  | B3_2 | CTGGAGGCAACCCTAAACTAC |
|  | B3_3 | GCGCAGCTGTTCCTTATAGT |
|  | B3_4 | CCAAGCTTGATAGGGTCTCTTAC |
|  | B3_5 | GGCTCCATGGTTGCACTAAA |
|  | B3_6 | CGGCAGCAGATGGTGATTA |
| Block 4 | B4_1 | TGCTACTGGTAGTGGGTATCT |
|  | B4_2 | CCCTGTTCGGTGTCATCTTT |
|  | B4_3 | ACGTGGGTCAGAGAGGTAAA |
|  | B4_4 | GGGCTTGTAATCCTGCTCTT |
|  | B4_5 | AGGGCCATATCTGCAAGAAC |
|  | B4_6 | CCAACTCATCCGCCAGTTTA |
|  | B4_7 | CAATGACAGCACCTTCACCC |
| Block 5 | B5_1 | GCCAACATGCTCTACCCTATAC |
|  | B5_2 | GGCAACGCCACAACATAAAG |
|  | B5_3 | CATGGAGTCAGTCGAGAAGAAG |
|  | B5_4 | TGCAGCACTGGAACACTATC |
|  | B5_5 | CACTTGGTCAGGCAGTAGTT |
|  | B5_6 | CCTACGAACGCCACCTATTC |
|  | B5_7 | ACCGCATGTACTCCTTCTTTAG |
|  | B5_8 | GCCGACGATGCTTCTGAATA |
|  | B5_9 | GGTAGGGTATAGAGCCAGAGTAG |
|  | B5_10 | GAAGTTCCACTCGTAGGTGTATG |
|  | B5_11 | ATGTCCAGCACACGGTTATC |
|  | B5_12 | AAAGGTGGTAGATGGCCTGG |
|  | B5 _13 | TGTCAACAGCCTGATTCCAC |
|  | B5_14 | GCCGAAATGTGGTGCAGATC |
| Block 6 | B6_1 | CCTGCATTACTACCGTCATCTC |
|  | B6_2 | CAAGCAACTCTACGGGCTATT |
|  | B6_3 | GTAGCCTGATTCGGGAGTTTAC |
|  | B6_4 | TTCCAGCCTTCACAGTCTATTT |
|  | B6_5 | CAATCAGAGGGCGAGGTATTC |
|  | B6_6 | CCAAGACTACTCAACCCGAATAA |
|  | B6_7 | CTTGCCTACCACTCTGACATAA |
|  | B6_8 | CCGGTCATTTCCTGCTCAATAC |
|  | B6_9 | CAGGCGCTGCTCTGTAATAA |
|  | B6_10 | CCCGCGCTCATGTAGTTTAT |
| Block 7 | B7_1 | CGGAGCTTACCTGCCTTTATC |
|  | B7_2 | GGGAGCGCGTTCACTTAATA |
|  | B7_3 | GACAGGAAACCGTGTGGAATA |
|  | B7_4 | ACTGGTAAGGCTGACTGTTATG |
|  | B7_5 | TACCGGGAGGTGGTGAATTA |
|  | B7_6 | CAAGCCCATCTCCTGCATTA |
|  | B7_7 | GGCTGTGCCTTCAGTAAGAT |
|  | B7_8 | CGCACCCTGATCTCACTTAAA |
|  | B7_9 | ACCTGATGTGTTTACCGAGTCT |
| All blocks | pJET2.1-F | CGACTCACTATAGGGAGAGCGGC |
|  | pJET2.1-R | AAGAACATCGATTTTCCATGGCAG |
