# Supplementary material for: Seamless assembly of recombinant adenoviral genomes from high-copy plasmids
Source: PLoS One. 2018 Jun 27;13(6):e0199563. doi: 10.1371/journal.pone.0199563 (PMC6021080; doi:10.1371/journal.pone.0199563)
Supplement: S5 Table — (DOCX) [file pone.0199563.s005.docx]

**S5 Table**

**Sequence variance from NCBI Reference Sequence: AC_000008.1**

| **Position in AC_000008.1** | **Reported** | **Observed** | **Gene** | **Consequence** |
| --- | --- | --- | --- | --- |
| 4952 | G | C | Iva2 | CAC→CAG, H163Q |
| 8783 | G | A | Pol | GCT→GTT, A4V |
| 11284 | T | C | 52K | TAC→CAC, Y79H |
| 14086 | - | A | Intergenic | Non-coding |
| 17387 | G | C | V | GTT→CGT, G282R |
| 20378 | T | C | Hexon | TTA→CTA, synonymous |
| 21163 | C | T | Hexon | TAC→TAT, synonymous |
| 21630 | G | A | Hexon | CCG→CAG, R930Q |
| 25995 | A | T | 100K | CCA→CCT, synonymous |
| 26741-26746 | GGCAGC | - | 22K | In-frame deletion of G183/S184 |
| 27161 | C | T | Intergenic | Non-coding |
| 27314 | C | A | pVIII | ATC→ATA, synonymous |
| 27339 | T | C | pVIII | TTG→CTG, synonymous |
| 27650-27651 | TC | CT | pVIII | CGTCCT→CGCTCT, P160S |
| 28120 | T | C | E3 12.5K | CTT→CCT, L88P |
| 30301-30303 | ACG | CGC | E3 RID-β | AAACGA→AACGCA, K80N, R81A |
| 30403-30404 | CA | AC | E3 RID-β | CCA→CCT, T116P |
| 34355* | - | A | Intergenic | Non-coding |
| 35776 | A | C | ITR | Unknown |

*Not observed in unamplified Ad5 reference DNA
