# Supplementary material for: Seamless assembly of recombinant adenoviral genomes from high-copy plasmids
Source: PLoS One. 2018 Jun 27;13(6):e0199563. doi: 10.1371/journal.pone.0199563 (PMC6021080; doi:10.1371/journal.pone.0199563)
Supplement: S6 Table — (DOCX) [file pone.0199563.s006.docx]

**S6 Table**

**Primers used to amplify E4 region from the packaged viral genome**

| Block | Primer | Sequence |
| --- | --- | --- |
| 7 | **Ad5-49** | GCGGGTTTCTGTCTCAAAAG |
| 7 | **Ad5-50** | TGCAGACCTGCACGATTATG |
