# Supplementary material for: Seamless assembly of recombinant adenoviral genomes from high-copy plasmids
Source: PLoS One. 2018 Jun 27;13(6):e0199563. doi: 10.1371/journal.pone.0199563 (PMC6021080; doi:10.1371/journal.pone.0199563)
Supplement: S1 Fig — (DOCX) [file pone.0199563.s007.docx]

**S1 Fig**

**Plasmid DNA sequences**

The numbers in parentheses correspond to the Ad5 reference sequence AC_00008.1 and represent the boundaries of each block. Recognition sites for BstBI are highlighted; for each plasmid, the BstBI site highlighted in red is the 5’ end with respect to the Ad5 reference sequence. Ad5 sequences are in upper case; vector sequences are shaded. Bases within the inserts that differ from the reference sequence are shown in lower case.

**Block 1 (1-3759)**

Plasmid name: pAd5-B1

Plasmid size: 6747 bp

Ad5 insert: 3759 bp

gcccctgcagccgaattatattatttttgccaaataatttttaacaaaagctctgaagtcttcttcatttaaattcttagatgatacttcatctggaaaattgtcccaattagtagcatcacgctgtgagtaagttctaaaccatttttttattgttgtattatctctaatcttactactcgatgagttttcggtattatctctatttttaacttggagcaggttccattcattgtttttttcatcatagtgaataaaatcaactgctttaacacttgtgcctgaacaccatatccatccggcgtaatacgactcactatagggagagcggccgccagatcttccggatggctcgagtttttcagcaagat**cttcgaaC**ATCATCAATAATATACCTTATTTTGGATTGAAGCCAATATGATAATGAGGGGGTGGAGTTTGTGACGTGGCGCGGGGCGTGGGAACGGGGCGGGTGACGTAGTAGTGTGGCGGAAGTGTGATGTTGCAAGTGTGGCGGAACACATGTAAGCGACGGATGTGGCAAAAGTGACGTTTTTGGTGTGCGCCGGTGTACACAGGAAGTGACAATTTTCGCGCGGTTTTAGGCGGATGTTGTAGTAAATTTGGGCGTAACCGAGTAAGATTTGGCCATTTTCGCGGGAAAACTGAATAAGAGGAAGTGAAATCTGAATAATTTTGTGTTACTCATAGCGCGTAATATTTGTCTAGGGCCGCGGGGACTTTGACCGTTTACGTGGAGACTCGCCCAGGTGTTTTTCTCAGGTGTTTTCCGCGTTCCGGGTCAAAGTTGGCGTTTTATTATTATAGTCAGCTGACGTGTAGTGTATTTATACCCGGTGAGTTCCTCAAGAGGCCACTCTTGAGTGCCAGCGAGTAGAGTTTTCTCCTCCGAGCCGCTCCGACACCGGGACTGAAAATGAGACATATTATCTGCCACGGAGGTGTTATTACCGAAGAAATGGCCGCCAGTCTTTTGGACCAGCTGATCGAAGAGGTACTGGCTGATAATCTTCCACCTCCTAGCCATTTTGAACCACCTACCCTTCACGAACTGTATGATTTAGACGTGACGGCCCCCGAAGATCCCAACGAGGAGGCGGTTTCGCAGATTTTTCCCGACTCTGTAATGTTGGCGGTGCAGGAAGGGATTGACTTACTCACTTTTCCGCCGGCGCCCGGTTCTCCGGAGCCGCCTCACCTTTCCCGGCAGCCCGAGCAGCCGGAGCAGAGAGCCTTGGGTCCGGTTTCTATGCCAAACCTTGTACCGGAGGTGATCGATCTTACCTGCCACGAGGCTGGCTTTCCACCCAGTGACGACGAGGATGAAGAGGGTGAGGAGTTTGTGTTAGATTATGTGGAGCACCCCGGGCACGGTTGCAGGTCTTGTCATTATCACCGGAGGAATACGGGGGACCCAGATATTATGTGTTCGCTTTGCTATATGAGGACCTGTGGCATGTTTGTCTACAGTAAGTGAAAATTATGGGCAGTGGGTGATAGAGTGGTGGGTTTGGTGTGGTAATTTTTTTTTTAATTTTTACAGTTTTGTGGTTTAAAGAATTTTGTATTGTGATTTTTTTAAAAGGTCCTGTGTCTGAACCTGAGCCTGAGCCCGAGCCAGAACCGGAGCCTGCAAGACCTACCCGCCGTCCTAAAATGGCGCCTGCTATCCTGAGACGCCCGACATCACCTGTGTCTAGAGAATGCAATAGTAGTACGGATAGCTGTGACTCCGGTCCTTCTAACACACCTCCTGAGATACACCCGGTGGTCCCGCTGTGCCCCATTAAACCAGTTGCCGTGAGAGTTGGTGGGCGTCGCCAGGCTGTGGAATGTATCGAGGACTTGCTTAACGAGCCTGGGCAACCTTTGGACTTGAGCTGTAAACGCCCCAGGCCATAAGGTGTAAACCTGTGATTGCGTGTGTGGTTAACGCCTTTGTTTGCTGAATGAGTTGATGTAAGTTTAATAAAGGGTGAGATAATGTTTAACTTGCATGGCGTGTTAAATGGGGCGGGGCTTAAAGGGTATATAATGCGCCGTGGGCTAATCTTGGTTACATCTGACCTCATGGAGGCTTGGGAGTGTTTGGAAGATTTTTCTGCTGTGCGTAACTTGCTGGAACAGAGCTCTAACAGTACCTCTTGGTTTTGGAGGTTTCTGTGGGGCTCATCCCAGGCAAAGTTAGTCTGCAGAATTAAGGAGGATTACAAGTGGGAATTTGAAGAGCTTTTGAAATCCTGTGGTGAGCTGTTTGATTCTTTGAATCTGGGTCACCAGGCGCTTTTCCAAGAGAAGGTCATCAAGACTTTGGATTTTTCCACACCGGGGCGCGCTGCGGCTGCTGTTGCTTTTTTGAGTTTTATAAAGGATAAATGGAGCGAAGAAACCCATCTGAGCGGGGGGTACCTGCTGGATTTTCTGGCCATGCATCTGTGGAGAGCGGTTGTGAGACACAAGAATCGCCTGCTACTGTTGTCTTCCGTCCGCCCGGCGATAATACCGACGGAGGAGCAGCAGCAGCAGCAGGAGGAAGCCAGGCGGCGGCGGCAGGAGCAGAGCCCATGGAACCCGAGAGCCGGCCTGGACCCTCGGGAATGAATGTTGTACAGGTGGCTGAACTGTATCCAGAACTGAGACGCATTTTGACAATTACAGAGGATGGGCAGGGGCTAAAGGGGGTAAAGAGGGAGCGGGGGGCTTGTGAGGCTACAGAGGAGGCTAGGAATCTAGCTTTTAGCTTAATGACCAGACACCGTCCTGAGTGTATTACTTTTCAACAGATCAAGGATAATTGCGCTAATGAGCTTGATCTGCTGGCGCAGAAGTATTCCATAGAGCAGCTGACCACTTACTGGCTGCAGCCAGGGGATGATTTTGAGGAGGCTATTAGGGTATATGCAAAGGTGGCACTTAGGCCAGATTGCAAGTACAAGATCAGCAAACTTGTAAATATCAGGAATTGTTGCTACATTTCTGGGAACGGGGCCGAGGTGGAGATAGATACGGAGGATAGGGTGGCCTTTAGATGTAGCATGATAAATATGTGGCCGGGGGTGCTTGGCATGGACGGGGTGGTTATTATGAATGTAAGGTTTACTGGCCCCAATTTTAGCGGTACGGTTTTCCTGGCCAATACCAACCTTATCCTACACGGTGTAAGCTTCTATGGGTTTAACAATACCTGTGTGGAAGCCTGGACCGATGTAAGGGTTCGGGGCTGTGCCTTTTACTGCTGCTGGAAGGGGGTGGTGTGTCGCCCCAAAAGCAGGGCTTCAATTAAGAAATGCCTCTTTGAAAGGTGTACCTTGGGTATCCTGTCTGAGGGTAACTCCAGGGTGCGCCACAATGTGGCCTCCGACTGTGGTTGCTTCATGCTAGTGAAAAGCGTGGCTGTGATTAAGCATAACATGGTATGTGGCAACTGCGAGGACAGGGCCTCTCAGATGCTGACCTGCTCGGACGGCAACTGTCACCTGCTGAAGACCATTCACGTAGCCAGCCACTCTCGCAAGGCCTGGCCAGTGTTTGAGCATAACATACTGACCCGCTGTTCCTTGCATTTGGGTAACAGGAGGGGGGTGTTCCTACCTTACCAATGCAATTTGAGTCACACTAAGATATTGCTTGAGCCCGAGAGCATGTCCAAGGTGAACCTGAACGGGGTGTTTGACATGACCATGAAGATCTGGAAGGTGCTGAGGTACGATGAGACCCGCACCAGGTGCAGACCCTGCGAGTGTGGCGGTAAACATATTAGGAACCAGCCTGTGATGCTGGATGTGACCGAGGAGCTGAGGCCCGATCACTTGGTGCTGGCCTGCACCCGCGCTGAGTTTGGCTCTAGCGATGAAGATACAGATTGAGGTACTGAAATGTGTGGGCGTGGCTTAAGGGTGGGAAAGAATATATAAGGTGGGGGTCTTATGTAGTTTTGTATCTGTTTTGCAGCAGCCGCCGCCGCCATGAGCACCAACTCGTTTGATGGAAGCATTGTGAGCTCATATTTGACAACGCGCATGCCCCCATGGGCCGGGGTGCGTCAGAATGTGATGGGCTCCAGCATTGATGGTCGCCCCGTCCTGCCCGCAAACTCTACTACCTTGACCTACGAGA**TTCGAAC**atctttctagaagatctcctacaatattctcagctgccatggaaaatcgatgttcttcttttattctctcaagattttcaggctgtatattaaaacttatattaagaactatgctaaccacctcatcaggaaccgttgtaggtggcgtgggttttcttggcaatcgactctcatgaaaactacgagctaaatattcaatatgttcctcttgaccaactttattctgcattttttttgaacgaggtttagagcaagcttcaggaaactgagacaggaattttattaaaaatttaaattttgaagaaagttcagggttaatagcatccattttttgctttgcaagttcctcagcattcttaacaaaagacgtctcttttgacatgtttaaagtttaaacctcctgtgtgaaattattatccgctcataattccacacattatacgagccggaagcataaagtgtaaagcctggggtgcctaatgagtgagctaactcacattaattgcgttgcgctcactgccaattgctttccagtcgggaaacctgtcgtgccagctgcattaatgaatcggccaacgcgcggggagaggcggtttgcgtattgggcgctcttccgcttcctcgctcactgactcgctgcgctcggtcgttcggctgcggcgagcggtatcagctcactcaaaggcggtaatacggttatccacagaatcaggggataacgcaggaaagaacatgtgagcaaaaggccagcaaaaggccaggaaccgtaaaaaggccgcgttgctggcgtttttccataggctccgcccccctgacgagcatcacaaaaatcgacgctcaagtcagaggtggcgaaacccgacaggactataaagataccaggcgtttccccctggaagctccctcgtgcgctctcctgttccgaccctgccgcttaccggatacctgtccgcctttctcccttcgggaagcgtggcgctttctcatagctcacgctgtaggtatctcagttcggtgtaggtcgttcgctccaagctgggctgtgtgcacgaaccccccgttcagcccgaccgctgcgccttatccggtaactatcgtcttgagtccaacccggtaagacacgacttatcgccactggcagcagccactggtaacaggattagcagagcgaggtatgtaggcggtgctacagagttcttgaagtggtggcctaactacggctacactagaaggacagtatttggtatctgcgctctgctgaagccagttaccttcggaaaaagagttggtagctcttgatccggcaaacaaaccaccgctggtagcggtggtttttttgtttgcaagcagcagattacgcgcagaaaaaaaggatctcaagaagatcctttgatcttttctacggggtctgacgctcagtggaacgaaaactcacgttaagggattttggtcatgagattatcaaaaaggatcttcacctagatccttttaaattaaaaatgaagttttaaatcaatctaaagtatatatgagtaaacttggtctgacagttaccaatgcttaatcagtgaggcacctatctcagcgatctgtctatttcgttcatccatagttgcctgactccccgtcgtgtagataactacgatacgggagggcttaccatctggccccagtgctgcaatgataccgcgagacccacgctcaccggctccagatttatcagcaataaaccagccagccggaagggccgagcgcagaagtggtcctgcaactttatccgcctccatccagtctattaattgttgccgggaagctagagtaagtagttcgccagttaatagtttgcgcaacgttgttgccattgctacaggcatcgtggtgtcacgctcgtcgtttggtatggcttcattcagctccggttcccaacgatcaaggcgagttacatgatcccccatgttgtgcaaaaaagcggttagctccttcggtcctccgatcgttgtcagaagtaagttggccgcagtgttatcactcatggttatggcagcactgcataattctcttactgtcatgccatccgtaagatgcttttctgtgactggtgagtactcaaccaagtcattctgagaatagtgtatgcggcgaccgagttgctcttgcccggcgtcaatacgggataataccgcgccacatagcagaactttaaaagtgctcatcattggaaaacgttcttcggggcgaaaactctcaaggatcttaccgctgttgagatccagttcgatgtaacccactcgtgcacccaactgatcttcagcatcttttactttcaccagcgtttctgggtgagcaaaaacaggaaggcaaaatgccgcaaaaaagggaataagggcgacacggaaatgttgaatactcatactcttcctttttcaatattattgaagcatttatcagggttattgtctcatgagcggatacatatttgaatgtatttagaaaaataaacaaataggggttccgcgcacatttccccgaaaagtgccacctgacgtctaagaaaccattattatcatgacattaacctataaaaataggcgtatcacgaggcc

**Block2 (3737-10881)**

Plasmid name: pAd5-B2

Plasmid size: 10133 bp

Ad5 insert: 7145 bp (in reverse orientation with respect to the Ad5 reference genome sequence)

gcccctgcagccgaattatattatttttgccaaataatttttaacaaaagctctgaagtcttcttcatttaaattcttagatgatacttcatctggaaaattgtcccaattagtagcatcacgctgtgagtaagttctaaaccatttttttattgttgtattatctctaatcttactactcgatgagttttcggtattatctctatttttaacttggagcaggttccattcattgtttttttcatcatagtgaataaaatcaactgctttaacacttgtgcctgaacaccatatccatccggcgtaatacgactcactatagggagagcggccgccagatcttccggatggctcgagtttttcagcaagat**GTTCGAA**CGAGCCACTTAATGCTTTCGCTTTCCAGCCTAACCGCTTACGCTGCGCGCGGCCAGTGGCCAAAAAAGCTAGCGCAGCAGCCGCCGCGCCTGGAAGGAAGCCAAAAGGAGCACTCCCCCGTTGTCTGACGTCGCACACCTGGGTTCGACACGCGGGCGGTAACCGCATGGATCACGGCGGACGGCCGGATACGGGGCTCGAACCCCGGTCGTCCGCCATGATACCCTTGCGAATTTATCCACCAGACCACGGAAGAGTGCCCGCTTACAGGCTCTCCTTTTGCACGGTCTAGAGCGTCAACGATTGCGCGCGCCTGACCGGCCAGAGCGTCCCGACCATGGAGCACTTTTTGCCGCTGCGCAACATCTGGAACCGCGTCCGCGACTTTCCGCGCGCCTCCACCACCGCCGCCGGCATCACCTGGATGTCCAGGTACATCTACGGATATCATCGCCTTATGTTGGAAGATCTCGCCCCCGGAGCCCCGGCCACCCTACGCTGGCCCCTCTACCGCCAGCCGCCGCCGCACTTTTTGGTGGGATACCAGTACCTGGTGCGGACTTGCAACGACTACGTATTTGACTCGAGGGCTTACTCGCGTCTCAGGTACACCGAGCTCTCGCAGCCGGGTCACCAGACCGTTAACTGGTCCGTTATGGCCAACTGCACTTACACCATCAACACGGGCGCATACCACCGCTTTGTGGACATGGATGACTTCCAGTCTACCCTCACGCAGGTGCAGCAGGCCATATTAGCCGAGCGCGTTGTCGCCGACCTAGCCCTGCTTCAGCCGATGAGGGGCTTCGGGGTCACACGCATGGGAGGAAGAGGGCGCCACCTACGGCCAAACTCCGCCGCCGCCGCAGCGATAGATGCAAGAGATGCAGGACAAGAGGAAGGAGAAGAAGAAGTGCCGGTAGAAAGGCTCATGCAAGACTACTACAAAGACCTGCGCCGATGTCAAAACGAAGCCTGGGGCATGGCCGACCGCCTGCGCATTCAGCAGGCCGGACCCAAGGACATGGTGCTTCTGTCGACCATCCGCCGTCTCAAGACCGCCTACTTTAATTACATCATCAGCAGCACCTCCGCCAGAAACAACCCCGACCGCCGCCCGCTGCCGCCCGCCACGGTGCTCAGCCTACCTTGCGACTGTGACTGGTTAGACGCCTTTCTCGAGAGGTTTTCCGATCCGGTCGATGCGGACTCGCTCAGGTCCCTCGGCGGCGGAGTACCTACACAACAATTGTTGAGATGCATCGTTAGCGCCGTATCCCTGCCGCATGGCAGCCCCCCGCCAACCCATAACCGGGACATGACGGGCGGCGTCTTCCAACTGCGCCCCCGCGAGAACGGCCGCGCCGTCACCGAGACCATGCGCCGTCGCCGCGGGGAGATGATCGAGCGCTTTGTCGACCGCCTCCCGGTGCGCCGTCGTCGCCGCCGTGTCCCCCCTCCCCCACCGCCGCCAGAAGAAGAAGAAGGGGAGGCCCTTATGGAAGAGGAGATTGAAGAAGAAGAAGAGGCCCCTGTAGCCTTTGAGCGCGAGGTGCGCGACACTGTCGCCGAGCTCATCCGTCTTCTGGAGGAGGAGTTAACCGTGTCGGCGCGCAACTCCCAGTTTTTCAACTTCGCCGTGGACTTCTACGAGGCCATGGAGCGCCTTGAGGCCTTGGGGGATATCAACGAATCCACGTTGCGACGCTGGGTTATGTACTTCTTCGTGGCAGAACACACCGCCACCACCCTCAACTACCTCTTTCAGCGCCTGCGAAACTACGCCGTCTTCGCCCGGCACGTGGAGCTCAATCTCGCGCAGGTGGTCATGCGCGCCCGCGATGCCGAAGGGGGCGTGGTCTACAGCCGCGTCTGGAACGAGGGAGGCCTCAACGCCTTCTCGCAGCTCATGGCCCGCATTTCCAACGACCTCGCCGCCACCGTGGAGCGAGCCGGACGCGGAGATCTCCAGGAGGAAGAGATCGAGCAGTTCATGGCCGAGATCGCCTATCAAGACAACTCAGGAGACGTGCAGGAGATTTTGCGCCAGGCCGCCGTCAACGACACCGAAATTGATTCTGTCGAACTCTCTTTCAGGtTCAAGCTCACCGGGCCCGTCGTCTTCACGCAGAGGCGCCAGATTCAGGAGATCAACCGCCGCGTCGTCGCGTTCGCCAGCAACCTACGCGCGCAGCACCAGCTCCTGCCCGCGCGCGGCGCCGACGTGCCCCTGCCCCCTCTCCCGGCGGGTCCGGAGCCCCCCCTACCTCCGGGGGCTCGCCCGCGTCACCGCTTTTAGATGCATCATCCAAGGACACCCCCGCGGCCCACCGCCCGCCGCGCGGTACCGTAGTCGCGCCGCGGGGATGCGGCCTCTTGCAAGCCATCGACGCCGCCACCAACCAGCCCCTGGAAATTAGGTATCACCTGGATCTAGCCCGCGCCCTGACCCGTCTATGCGAGGTAAACCTGCAGGAGCTCCCGCCTGACCTGACGCCGCGGGAGCTCCAGACCATGGACAGCTCCCATCTGCGCGATGTTGTCATCAAGCTCCGACCGCCGCGCGCGGACATCTGGACTTTGGGCTCGCGCGGCGTGGTGGTCCGATCCACCGTAACTCCCCTCGAGCAGCCAGACGGTCAAGGACAAGCAGCCGAAGTAGAAGACCACCAGCCAAACCCGCCAGGCGAGGGGCTCAAATTCCCACTCTGCTTCCTTGTGCGCGGTCGTCAGGTCAACCTCGTGCAGGATGTACAGCCCGTGCACCGCTGCCAGTACTGCGCACGTTTTTACAAAAGCCAGCACGAGTGTTCGGCCCGTCGCAGGGACTTCTACTTTCACCACATCAATAGCCACTCCTCCAATTGGTGGCGGGAGATCCAGTTCTTCCCGATCGGCTCGCATCCTCGCACCGAGCGTCTCTTTGTCACCTACGATGTAGAGACCTATACTTGGATGGGGGCCTTTGGGAAGCAGCTCGTGCCCTTCATGCTGGTCATGAAGTTCGGCGGAGATGAGCCTCTAGTGACTGCCGCGCGAGACCTAGCCGCGAACCTTGGATGGGACCGCTGGGAACAAGACCCGCTTACCTTCTACTGCATCACCCCAGAAAAAATGGCCATAGGTCGCCAGTTTAGGACCTTTCGCGACCACCTGCAAATGCTAATGGCCCGTGACCTGTGGAGCTCATTCGTCGCTTCCAACCCTCATCTTGCAGACTGGGCCCTTTCAGAGCACGGGCTCAGCTCCCCTGAAGAGCTCACCTACGAGGAACTTAAAAAATTGCCTTCCATCAAGGGCATCCCGCGCTTCTTGGAACTTTACATTGTGGGCCACAACATCAACGGCTTTGACGAGATCGTGCTCGCCGCCCAGGTAATTAACAACCGTTCCGAGGTGCCGGGACCCTTCCGCATCACACGCAACTTTATGCCTCGCGCGGGAAAGATACTCTTCAACGATGTCACCTTCGCCCTGCCAAATCCGCGTTCCAAAAAGCGCACGGACTTTTTGCTCTGGGAGCAGGGCGGATGCGACGACACTGACTTCAAATACCAGTACCTCAAAGTCATGGTCAGGGACACCTTTGCGCTCACCCACACCTCGCTCCGGAAGGCCGCGCAGGCATACGCGCTACCCGTAGAAAAGGGATGCTGCGCCTACCAGGCCGTCAACCAGTTCTACATGCTAGGCTCTTACCGTTCGGAGGCCGACGGGTTTCCGATCCAAGAGTACTGGAAAGACCGCGAAGAGTTTGTCCTCAACCGCGAGCTGTGGAAAAAAAAGGGACAGGATAAGTATGACATCATCAAGGAAACCCTGGACTACTGCGCCCTAGACGTGCAGGTCACCGCCGAGCTGGTCAACAAGCTGCGCGACTCCTACGCCTCCTTCGTGCGTGACGCGGTAGGTCTCACAGACGCCAGCTTCAACGTCTTCCAGCGTCCAACCATATCATCCAACTCACATGCCATCTTCAGGCAGATAGTCTTCCGAGCAGAGCAGCCCGCCCGTAGCAACCTCGGTCCCGACCTCCTCGCTCCCTCGCACGAACTATACGATTACGTGCGCGCCAGCATCCGCGGTGGAAGATGCTACCCTACATATCTTGGAATACTCAGAGAGCCCCTCTACGTTTACGACATTTGCGGCATGTACGCCTCCGCGCTCACCCACCCCATGCCATGGGGTCCCCCACTCAACCCATACGAGCGCGCGCTTGCCGCCCGCGCATGGCAGCAGGCGCTAGACTTGCAAGGATGCAAGATAGACTACTTCGACGCGCGCCTGCTGCCCGGGGTCTTTACCGTGGACGCAGACCCCCCGGACGAGACGCAGCTAGACCCCCTACCGCCATTCTGCTCGCGCAAGGGCGGCCGCCTCTGCTGGACCAACGAGCGCCTACGCGGAGAGGTAGCCACCAGCGTTGACCTTGTCACCCTGCACAACCGCGGTTGGCGCGTGCACCTGGTGCCCGACGAGCGCACCACCGTCTTTCCCGAATGGCGGTGCGTTGCGCGCGAATACGTGCAGCTAAACATCGCGGCCAAGGAGCGCGCCGATCGCGACAAAAACCAAACCCTGCGCTCCATCGCCAAGTTGCTGTCCAACGCCCTCTACGGGTCGTTTGCCACCAAGCTTGACAACAAAAAGATTGTCTTTTCTGACCAGATGGATGCGGCCACCCTCAAAGGCATCACCGCGGGCCAGGTGAATATCAAATCCTCCTCGTTTTTGGAAACTGACAATCTTAGCGCAGAAGTCATGCCCGCTTTTCAGAGGGAGTACTCACCCCAACAGCTGGCCCTCGCAGACAGCGATGCGGAAGAGAGTGAGGACGAACGCGCCCCCACCCCCTTTTATAGCCCCCCTTCAGGAACACCCGGTCACGTGGCCTACACCTACAAACCAATCACCTTCCTTGATGCCGAAGAGGGCGACATGTGTCTTCACACCCTGGAGCGAGTGGACCCCCTAGTGGACAACGACCGCTACCCCTCCCACTTAGCCTCCTTCGTGCTGGCCTGGACGCGAGCCTTTGTCTCAGAGTGGTCCGAGTTTCTATACGAGGAGGACCGCGGAACACCGCTCGAGGACAGGCCTCTCAAGTCTGTATACGGGGACACGGACAGCCTTTTCGTCACCGAGCGTGGACACCGGCTCATGGAAACCAGAGGTAAGAAACGCATCAAAAAGCATGGGGGAAACCTGGTTTTTGACCCCGAACGGCCAGAGCTCACCTGGCTCGTGGAATGCGAGACCGTCTGCGGGGCCTGCGGCGCGGATGCCTACTCCCCGGAATCGGTATTTCTCGCGCCCAAGCTCTACGCCCTCAAAAGTCTGCACTGCCCCTCGTGCGGCGCCTCCTCCAAGGGCAAGCTGCGCGCCAAGGGCCACGCCGCGGAGGGGCTGGACTATGACACCATGGTCAAATGCTACCTGGCCGACGCGCAGGGCGAAGACCGGCAGCGCTTCAGCACCAGCAGGACCAGCCTCAAGCGCACCCTGGCCAGCGCGCAGCCCGGAGCGCACCCCTTCACCGTGACCCAGACTACGCTGACGAGGACCCTGCGCCCGTGGAAAGACATGACCCTGGCCCGTCTGGACGAGCACCGACTACTGCCGTACAGCGAAAGCCGCCCCAACCCGCGAAACGAGGAGATATGCTGGATCGAGATGCCGTAGAGCAGGTGACCGAGCTGTGGGACCGCCTGGAACTGCTTGGTCAAACGCTCAAAAGCATGCCTACGGCGGACGGTCTCAAACCGTTGAAAAACTTTGCTTCCTTGCAAGAACTGCTATCGCTGGGCGGCGAGCGCCTTCTGGCGGATTTGGTCAGGGAAAACATGCGAGTCAGGGACATGCTTAACGAAGTGGCCCCCCTGCTCAGGGATGACGGCAGCTGCAGCTCTCTTAACTACCAGTTGCAgCCGGTAATAGGTGTGATTTACGGGCCCACCGGCTGCGGTAAGTCGCAGCTGCTCAGGAACCTGCTTTCTTCCCAGCTGATCTCCCCTACCCCGGAAACCGTTTTCTTCATCGCCCCGCAGGTAGACATGATCCCCCCATCTGAACTCAAAGCGTGGGAAATGCAAATCTGTGAGGGTAACTACGCCCCTGGGCCGGATGGAACCATTATACCGCAGTCTGGCACCCTCCGCCCGCGCTTTGTAAAAATGGCCTATGACGATCTCATCCTGGAACACAACTATGACGTTAGTGATCCCAGAAATATCTTCGCCCAGGCCGCCGCCCGTGGGCCCATTGCCATCATTATGGACGAATGCATGGAAAATCTTGGAGGTCACAAGGGCGTCTCCAAGTTCTTCCACGCATTTCCTTCTAAGCTACATGACAAATTTCCCAAGTGCACCGGATACACTGTGCTGGTGGTTCTGCACAACATGAATCCCCGGAGGGATATGGCTGGGAACATAGCCAACCTAAAAATACAGTCCAAGATGCATCTCATATCCCCACGTATGCACCCATCCCAGCTTAACCGCTTTGTAAACACTTACACCAAGGGCCTGCCCCTGGCAATCAGCTTGCTACTGAAAGACATTTTTAGGCACCACGCCCAGCGCTCCTGCTACGACTGGATCATCTACAACACCACCCCGCAGCATGAAGCTCTGCAGTGGTGCTACCTCCACCCCAGAGACGGGCTTATGCCCATGTATCTGAACATCCAGAGTCACCTTTACCACGTCCTGGAAAAAATACACAGGACCCTCAACGACCGAGACCGCTGGTCCCGGGCCTACCGCGCGCGCAAAACCCCTAAATAAAGACAGCAAGACACTTGCTTGATCCAAATCCAAACAGAGTCTGGTTTTTTATTTATGTTTTAAACCGCATTGGGAGGGGAGGAAGCCTTCAGGGCAGAAACCTGCTGGCGCAGATCCAACAGCTGCTGAGAAACGACATTAAGTTCCCGGGTCAAAGAATCCAATTGTGCCAAAAGAGCCGTCAACTTGTCATCGCGGGCGGATGAACGGGAAGCTGCACTGCTTGCAAGCGGGCTCAGGAAAGCAAAGTCAGTCACAATCCCGCGGGCGGTGGCTGCAGCGGCTGAAGCGGCGGCGGAGGCTGCAGTCTCCAACGGCGTTCCAGACACGGTCTCGTAGGTCAAGGTAGTAGAGTTCGAAGatctttctagaagatctcctacaatattctcagctgccatggaaaatcgatgttcttcttttattctctcaagattttcaggctgtatattaaaacttatattaagaactatgctaaccacctcatcaggaaccgttgtaggtggcgtgggttttcttggcaatcgactctcatgaaaactacgagctaaatattcaatatgttcctcttgaccaactttattctgcattttttttgaacgaggtttagagcaagcttcaggaaactgagacaggaattttattaaaaatttaaattttgaagaaagttcagggttaatagcatccattttttgctttgcaagttcctcagcattcttaacaaaagacgtctcttttgacatgtttaaagtttaaacctcctgtgtgaaattattatccgctcataattccacacattatacgagccggaagcataaagtgtaaagcctggggtgcctaatgagtgagctaactcacattaattgcgttgcgctcactgccaattgctttccagtcgggaaacctgtcgtgccagctgcattaatgaatcggccaacgcgcggggagaggcggtttgcgtattgggcgctcttccgcttcctcgctcactgactcgctgcgctcggtcgttcggctgcggcgagcggtatcagctcactcaaaggcggtaatacggttatccacagaatcaggggataacgcaggaaagaacatgtgagcaaaaggccagcaaaaggccaggaaccgtaaaaaggccgcgttgctggcgtttttccataggctccgcccccctgacgagcatcacaaaaatcgacgctcaagtcagaggtggcgaaacccgacaggactataaagataccaggcgtttccccctggaagctccctcgtgcgctctcctgttccgaccctgccgcttaccggatacctgtccgcctttctcccttcgggaagcgtggcgctttctcatagctcacgctgtaggtatctcagttcggtgtaggtcgttcgctccaagctgggctgtgtgcacgaaccccccgttcagcccgaccgctgcgccttatccggtaactatcgtcttgagtccaacccggtaagacacgacttatcgccactggcagcagccactggtaacaggattagcagagcgaggtatgtaggcggtgctacagagttcttgaagtggtggcctaactacggctacactagaaggacagtatttggtatctgcgctctgctgaagccagttaccttcggaaaaagagttggtagctcttgatccggcaaacaaaccaccgctggtagcggtggtttttttgtttgcaagcagcagattacgcgcagaaaaaaaggatctcaagaagatcctttgatcttttctacggggtctgacgctcagtggaacgaaaactcacgttaagggattttggtcatgagattatcaaaaaggatcttcacctagatccttttaaattaaaaatgaagttttaaatcaatctaaagtatatatgagtaaacttggtctgacagttaccaatgcttaatcagtgaggcacctatctcagcgatctgtctatttcgttcatccatagttgcctgactccccgtcgtgtagataactacgatacgggagggcttaccatctggccccagtgctgcaatgataccgcgagacccacgctcaccggctccagatttatcagcaataaaccagccagccggaagggccgagcgcagaagtggtcctgcaactttatccgcctccatccagtctattaattgttgccgggaagctagagtaagtagttcgccagttaatagtttgcgcaacgttgttgccattgctacaggcatcgtggtgtcacgctcgtcgtttggtatggcttcattcagctccggttcccaacgatcaaggcgagttacatgatcccccatgttgtgcaaaaaagcggttagctccttcggtcctccgatcgttgtcagaagtaagttggccgcagtgttatcactcatggttatggcagcactgcataattctcttactgtcatgccatccgtaagatgcttttctgtgactggtgagtactcaaccaagtcattctgagaatagtgtatgcggcgaccgagttgctcttgcccggcgtcaatacgggataataccgcgccacatagcagaactttaaaagtgctcatcattggaaaacgttcttcggggcgaaaactctcaaggatcttaccgctgttgagatccagttcgatgtaacccactcgtgcacccaactgatcttcagcatcttttactttcaccagcgtttctgggtgagcaaaaacaggaaggcaaaatgccgcaaaaaagggaataagggcgacacggaaatgttgaatactcatactcttcctttttcaatattattgaagcatttatcagggttattgtctcatgagcggatacatatttgaatgtatttagaaaaataaacaaataggggttccgcgcacatttccccgaaaagtgccacctgacgtctaagaaaccattattatcatgacattaacctataaaaataggcgtatcacgaggcc

**Block 3** (10862-14499)

Plasmid name: pAd5-B3

Plasmid size: 6626 bp

Ad5 insert: 3638 bp

gcccctgcagccgaattatattatttttgccaaataatttttaacaaaagctctgaagtcttcttcatttaaattcttagatgatacttcatctggaaaattgtcccaattagtagcatcacgctgtgagtaagttctaaaccatttttttattgttgtattatctctaatcttactactcgatgagttttcggtattatctctatttttaacttggagcaggttccattcattgtttttttcatcatagtgaataaaatcaactgctttaacacttgtgcctgaacaccatatccatccggcgtaatacgactcactatagggagagcggccgccagatcttccggatggctcgagtttttcagcaagatcttcgaaCGAAAGCATTAAGTGGCTCGCTCCCTGTAGCCGGAGGGTTATTTTCCAAGGGTTGAGTCGCGGGACCCCCGGTTCGAGTCTCGGACCGGCCGGACTGCGGCGAACGGGGGTTTGCCTCCCCGTCATGCAAGACCCCGCTTGCAAATTCCTCCGGAAACAGGGACGAGCCCCTTTTTTGCTTTTCCCAGATGCATCCGGTGCTGCGGCAGATGCGCCCCCCTCCTCAGCAGCGGCAAGAGCAAGAGCAGCGGCAGACATGCAGGGCACCCTCCCCTCCTCCTACCGCGTCAGGAGGGGCGACATCCGCGGTTGACGCGGCAGCAGATGGTGATTACGAACCCCCGCGGCGCCGGGCCCGGCACTACCTGGACTTGGAGGAGGGCGAGGGCCTGGCGCGGCTAGGAGCGCCCTCTCCTGAGCGGcACCCAAGGGTGCAGCTGAAGCGTGATACGCGTGAGGCGTACGTGCCGCGGCAGAACCTGTTTCGCGACCGCGAGGGAGAGGAGCCCGAGGAGATGCGGGATCGAAAGTTCCACGCAGGGCGCGAGCTGCGGCATGGCCTGAATCGCGAGCGGTTGCTGCGCGAGGAGGACTTTGAGCCCGACGCGCGAACCGGGATTAGTCCCGCGCGCGCACACGTGGCGGCCGCCGACCTGGTAACCGCATACGAGCAGACGGTGAACCAGGAGATTAACTTTCAAAAAAGCTTTAACAACCACGTGCGTACGCTTGTGGCGCGCGAGGAGGTGGCTATAGGACTGATGCATCTGTGGGACTTTGTAAGCGCGCTGGAGCAAAACCCAAATAGCAAGCCGCTCATGGCGCAGCTGTTCCTTATAGTGCAGCACAGCAGGGACAACGAGGCATTCAGGGATGCGCTGCTAAACATAGTAGAGCCCGAGGGCCGCTGGCTGCTCGATTTGATAAACATCCTGCAGAGCATAGTGGTGCAGGAGCGCAGCTTGAGCCTGGCTGACAAGGTGGCCGCCATCAACTATTCCATGCTTAGCCTGGGCAAGTTTTACGCCCGCAAGATATACCATACCCCTTACGTTCCCATAGACAAGGAGGTAAAGATCGAGGGGTTCTACATGCGCATGGCGCTGAAGGTGCTTACCTTGAGCGACGACCTGGGCGTTTATCGCAACGAGCGCATCCACAAGGCCGTGAGCGTGAGCCGGCGGCGCGAGCTCAGCGACCGCGAGCTGATGCACAGCCTGCAAAGGGCCCTGGCTGGCACGGGCAGCGGCGATAGAGAGGCCGAGTCCTACTTTGACGCGGGCGCTGACCTGCGCTGGGCCCCAAGCCGACGCGCCCTGGAGGCAGCTGGGGCCGGACCTGGGCTGGCGGTGGCACCCGCGCGCGCTGGCAACGTCGGCGGCGTGGAGGAATATGACGAGGACGATGAGTACGAGCCAGAGGACGGCGAGTACTAAGCGGTGATGTTTCTGATCAGATGATGCAAGACGCAACGGACCCGGCGGTGCGGGCGGCGCTGCAGAGCCAGCCGTCCGGCCTTAACTCCACGGACGACTGGCGCCAGGTCATGGACCGCATCATGTCGCTGACTGCGCGCAATCCTGACGCGTTCCGGCAGCAGCCGCAGGCCAACCGGCTCTCCGCAATTCTGGAAGCGGTGGTCCCGGCGCGCGCAAACCCCACGCACGAGAAGGTGCTGGCGATCGTAAACGCGCTGGCCGAAAACAGGGCCATCCGGCCCGACGAGGCCGGCCTGGTCTACGACGCGCTGCTTCAGCGCGTGGCTCGTTACAACAGCGGCAACGTGCAGACCAACCTGGACCGGCTGGTGGGGGATGTGCGCGAGGCCGTGGCGCAGCGTGAGCGCGCGCAGCAGCAGGGCAACCTGGGCTCCATGGTTGCACTAAACGCCTTCCTGAGTACACAGCCCGCCAACGTGCCGCGGGGACAGGAGGACTACACCAACTTTGTGAGCGCACTGCGGCTAATGGTGACTGAGACACCGCAAAGTGAGGTGTACCAGTCTGGGCCAGACTATTTTTTCCAGACCAGTAGACAAGGCCTGCAGACCGTAAACCTGAGCCAGGCTTTCAAAAACTTGCAGGGGCTGTGGGGGGTGCGGGCTCCCACAGGCGACCGCGCGACCGTGTCTAGCTTGCTGACGCCCAACTCGCGCCTGTTGCTGCTGCTAATAGCGCCCTTCACGGACAGTGGCAGCGTGTCCCGGGACACATACCTAGGTCACTTGCTGACACTGTACCGCGAGGCCATAGGTCAGGCGCATGTGGACGAGCATACTTTCCAGGAGATTACAAGTGTCAGCCGCGCGCTGGGGCAGGAGGACACGGGCAGCCTGGAGGCAACCCTAAACTACCTGCTGACCAACCGGCGGCAGAAGATCCCCTCGTTGCACAGTTTAAACAGCGAGGAGGAGCGCATTTTGCGCTACGTGCAGCAGAGCGTGAGCCTTAACCTGATGCGCGACGGGGTAACGCCCAGCGTGGCGCTGGACATGACCGCGCGCAACATGGAACCGGGCATGTATGCCTCAAACCGGCCGTTTATCAACCGCCTAATGGACTACTTGCATCGCGCGGCCGCCGTGAACCCCGAGTATTTCACCAATGCCATCTTGAACCCGCACTGGCTACCGCCCCCTGGTTTCTACACCGGGGGATTCGAGGTGCCCGAGGGTAACGATGGATTCCTCTGGGACGACATAGACGACAGCGTGTTTTCCCCGCAACCGCAGACCCTGCTAGAGTTGCAACAGCGCGAGCAGGCAGAGGCGGCGCTGCGAAAGGAAAGCTTCCGCAGGCCAAGCAGCTTGTCCGATCTAGGCGCTGCGGCCCCGCGGTCAGATGCTAGTAGCCCATTTCCAAGCTTGATAGGGTCTCTTACCAGCACTCGCACCACCCGCCCGCGCCTGCTGGGCGAGGAGGAGTACCTAAACAACTCGCTGCTGCAGCCGCAGCGCGAAAAAAACCTGCCTCCGGCATTTCCCAACAACGGGATAGAGAGCCTAGTGGACAAGATGAGTAGATGGAAGACGTACGCGCAGGAGCACAGGGACGTGCCAGGCCCGCGCCCGCCCACCCGTCGTCAAAGGCACGACCGTCAGCGGGGTCTGGTGTGGGAGGACGATGACTCGGCAGACGACAGCAGCGTCCTGGATTTGGGAGGGAGTGGCAACCCGTTTGCGCACCTTCGCCCCAGGCTGGGGAGAATGTTTTAAAAAAAAAAAAaGCATGATGCAAAATAAAAAACTCACCAAGGCCATGGCACCGAGCGTTGGTTTTCTTGTATTCCCCTTAGTATGCGGCGCGCGGCGATGTATGAGGAAGGTCCTCCTCCCTCCTACGAGAGTGTGGTGAGCGCGGCGCCAGTGGCGGCGGCGCTGGGTTCTCCCTTCGATGCTCCCCTGGACCCGCCGTTTGTGCCTCCGCGGTACCTGCGGCCTACCGGGGGGAGAAACAGCATCCGTTACTCTGAGTTGGCACCCCTATTCGACACCACCCGTGTGTACCTGGTGGACAACAAGTCAACGGATGTGGCATCCCTGAACTACCAGAACGACCACAGCAACTTTCTGACCACGGTCATTCAAAACAATGACTACAGCCCGGGGGAGGCAAGCACACAGACCATCAATCTTGACGATTCGAACatctttctagaagatctcctacaatattctcagctgccatggaaaatcgatgttcttcttttattctctcaagattttcaggctgtatattaaaacttatattaagaactatgctaaccacctcatcaggaaccgttgtaggtggcgtgggttttcttggcaatcgactctcatgaaaactacgagctaaatattcaatatgttcctcttgaccaactttattctgcattttttttgaacgaggtttagagcaagcttcaggaaactgagacaggaattttattaaaaatttaaattttgaagaaagttcagggttaatagcatccattttttgctttgcaagttcctcagcattcttaacaaaagacgtctcttttgacatgtttaaagtttaaacctcctgtgtgaaattattatccgctcataattccacacattatacgagccggaagcataaagtgtaaagcctggggtgcctaatgagtgagctaactcacattaattgcgttgcgctcactgccaattgctttccagtcgggaaacctgtcgtgccagctgcattaatgaatcggccaacgcgcggggagaggcggtttgcgtattgggcgctcttccgcttcctcgctcactgactcgctgcgctcggtcgttcggctgcggcgagcggtatcagctcactcaaaggcggtaatacggttatccacagaatcaggggataacgcaggaaagaacatgtgagcaaaaggccagcaaaaggccaggaaccgtaaaaaggccgcgttgctggcgtttttccataggctccgcccccctgacgagcatcacaaaaatcgacgctcaagtcagaggtggcgaaacccgacaggactataaagataccaggcgtttccccctggaagctccctcgtgcgctctcctgttccgaccctgccgcttaccggatacctgtccgcctttctcccttcgggaagcgtggcgctttctcatagctcacgctgtaggtatctcagttcggtgtaggtcgttcgctccaagctgggctgtgtgcacgaaccccccgttcagcccgaccgctgcgccttatccggtaactatcgtcttgagtccaacccggtaagacacgacttatcgccactggcagcagccactggtaacaggattagcagagcgaggtatgtaggcggtgctacagagttcttgaagtggtggcctaactacggctacactagaaggacagtatttggtatctgcgctctgctgaagccagttaccttcggaaaaagagttggtagctcttgatccggcaaacaaaccaccgctggtagcggtggtttttttgtttgcaagcagcagattacgcgcagaaaaaaaggatctcaagaagatcctttgatcttttctacggggtctgacgctcagtggaacgaaaactcacgttaagggattttggtcatgagattatcaaaaaggatcttcacctagatccttttaaattaaaaatgaagttttaaatcaatctaaagtatatatgagtaaacttggtctgacagttaccaatgcttaatcagtgaggcacctatctcagcgatctgtctatttcgttcatccatagttgcctgactccccgtcgtgtagataactacgatacgggagggcttaccatctggccccagtgctgcaatgataccgcgagacccacgctcaccggctccagatttatcagcaataaaccagccagccggaagggccgagcgcagaagtggtcctgcaactttatccgcctccatccagtctattaattgttgccgggaagctagagtaagtagttcgccagttaatagtttgcgcaacgttgttgccattgctacaggcatcgtggtgtcacgctcgtcgtttggtatggcttcattcagctccggttcccaacgatcaaggcgagttacatgatcccccatgttgtgcaaaaaagcggttagctccttcggtcctccgatcgttgtcagaagtaagttggccgcagtgttatcactcatggttatggcagcactgcataattctcttactgtcatgccatccgtaagatgcttttctgtgactggtgagtactcaaccaagtcattctgagaatagtgtatgcggcgaccgagttgctcttgcccggcgtcaatacgggataataccgcgccacatagcagaactttaaaagtgctcatcattggaaaacgttcttcggggcgaaaactctcaaggatcttaccgctgttgagatccagttcgatgtaacccactcgtgcacccaactgatcttcagcatcttttactttcaccagcgtttctgggtgagcaaaaacaggaaggcaaaatgccgcaaaaaagggaataagggcgacacggaaatgttgaatactcatactcttcctttttcaatattattgaagcatttatcagggttattgtctcatgagcggatacatatttgaatgtatttagaaaaataaacaaataggggttccgcgcacatttccccgaaaagtgccacctgacgtctaagaaaccattattatcatgacattaacctataaaaataggcgtatcacgaggcc

**Block 4 (14475-18018)**

Plasmid name pAd5-B4

Plasmid size: 6532 bp

Ad5 insert: 3544 bp (in reverse orientation with respect to the Ad5 reference genome sequence)

gcccctgcagccgaattatattatttttgccaaataatttttaacaaaagctctgaagtcttcttcatttaaattcttagatgatacttcatctggaaaattgtcccaattagtagcatcacgctgtgagtaagttctaaaccatttttttattgttgtattatctctaatcttactactcgatgagttttcggtattatctctatttttaacttggagcaggttccattcattgtttttttcatcatagtgaataaaatcaactgctttaacacttgtgcctgaacaccatatccatccggcgtaatacgactcactatagggagagcggccgccagatcttccggatggctcgagtttttcagcaagatGTTCGAAAGTTGATGTCTTCCATTCTACAAAATAGTTACAGGACCAAGCGAGCGTGAGAGTCCAGACTTTTTATTTTGATTTTTCCACATGCAACTTGTTTTTAATCAGTGTCTCTGCGCCTGCAAGGCCACGGATGCAATTCCGGGCACGGCGCCAATCGCCGCGGCGATCAGTGGAATAAGGAGGGGCAGGATACCGCCGCGCATGCGACGGTGCGACGCGCGCCGCCGCCGGTGGTGCGCACGACGCATGCCGCCCGTCAGGCCGTGGCCGGCCATGCCCCTCCTACGGTGCATTCTTCCTCGGAATCCCGGCACCGGGAAACGGAGGCGGCAGGTGAGGGCCATATCTGCAAGAACCACAAAGACCGGCTTTTAAACGATGCTGGGGTGGTAGCGCGCTGTTGGCAGCACCAGGGTCCTGCCTCCTTCGCGAGCCACCCTGCGCACGGAAATCGGGGCCAGCACGGGCTGGCGACGGCGACGGCGGCGGCGGGTTCCAGTGGTGGTTCGGCGTCGGGTAGTTGCTCGTCTTCTGGGGCGGTAGGTGTAGCCACGATAGCCGGGGGTAGGCGCAATGGAAGGATGTAGGGCATATTCGGGCAGTAGCGCGCTGGCGGCGCCGTACTTCCTCGAAgCGCGCGGGCGCCGGGGGGCTGAAACGCGAAACATCCACGGGTCCGTTTGCACCTCCGTAGAGGTCTTGGACGCGGCCGCAGCGACCGCCTGCACCGCGGCATCCGCCACCGCTGAGGCAACCGGGGACGTTTGTGTCTCCATGCCCTCTGTGGCGGTGGCAATACTGGTGCTACTGGTAGTGGGTATCTGAACGTCCACGGTCTGCACGCCCAGTCCCGGCGCCACCTGCTTGATTGGCCGCACGCGGACCTCGGGCTCCAGCCCAGGTTCCACGGTCATTTTTTCCAAGACATCTTCCAGTCGCTGGCGCTTGGGTACCATCAGCTGCACGGTGGGTGCCAAGTCACCAGACTCGCGCTTTAGGCCGCGCTTTTCTTCGGACGGTGCAAGCGCGGGCAGCACCTGCTGCAGTGTTACGGGCTTTAGGCTAGGTGTTGGGTTGCCCTCGTCCAGCGGCAACGCCAGCATGTCCTTATGCCGCTTTCCGTAGGCAAACTCCCCGAGGCGCTCGTTGGCCTGCTCAAGCAGGTCCTCGTCGCCGTACACCTCATCATACACGCGCTTGTAGGTGCGGGTGGAGCGCTCACCGGGCGTAAAGACTACGGTGGTGCCGGGTCGCAAAACACGTTTTACGCGTCGACCTTTCCACTGTACCCGTCGCCTGGGCGCGGTAGCGTGCAGCAGTTCCACCTCGTCGTCAAGTTCATCATCATCATCTTTCTTTTTCTTTTTGACCCGCTTTAGCTTTCGGGGCTTGTAATCCTGCTCTTCCTTCTTCGGGGGGCCATAGATCTCCGGCGCGATGACCTGGAGCATCTCTTCTTTGATTTTGCGCTTGGACATAGCTTCGTTGCGCGCCGCCGCCGCTGGATACATACAACAGTACGAGTCTAAGTAGTTTTTTCTTGCAATCTAGTTGCGCGGGGGGCGGGTGCGCACGGGCACGCGCAGGCCGCTAACCGAGTCGCGCACCCAATACACGTTGCCCCTGCGACCCTGAGTCATAGCACTAATGGCCGCGGCTGCTGCGGCGGCCGCTCGTCGCCTGGACCTGGGGGGCACAGTGACAATACCCGCGGCCAGCCTTCGAGCGGCCCGCATGGCCGCCCGTCGGCCGGTGCGACGTGCGCGGTTAAGCAGGGCCGCCGCCGCGCGTTGGGCGGCAGTGCCGGGTCGGCGGCGGTGGCGACGTGCTACGCGCCTCCGCCGTCTCTTCATTTTAGCATAGCGCCGGGCTCCGCGCACCACGGTCTGAATGGCCGCGTCCACTGTGGACACTGGTGGCGGCGTGGGCGTGTAGTTGCGCGCCTCCTCCACCACCGCGTCGATGGCGTCATCGACGGTGGTGCGCCCAGTGCGGCCGCGTTTGTGCGCGCCCCAGGGCGCGCGGTAGTGCCCGCGCACGCGCACTGGGTGTTGGTCGGAGCGCTTCTTGGCCCCGCCAAACATCTTGCTTGGGAAGCGCAGGCCCCAGCCTGTGTTATTGCTGGGCGATATAAGGATGGACATGCTTGCTCAAAAAGTGCGGCTCGATAGGACGCGCGGCGAGACTATGCCCAGGGCCTTGTAAACGTAGGGGCAGGTGCGGCGTCTGGCGTCAGTAATGGTCACTCGCTGGACTCCTCCGATGCTGTTGCGCAGCGGTAGCGTCCCGTGATCTGTGAGAGCAGGAACGTTTTCACTGACGGTGGTGATGGTGGGGGCTGGCGGGCGCGCCAAAATCTGGTTCTCGGGAAAGCGATTGAACACGTGGGTCAGAGAGGTAAACTGGCGGATGAGTTGGGAGTAGACGGCCTGGTCGTTGTAGAAGCTCTTGGAGTGCACGGGCAACAGCTCGGCGCCCACCACCGGAAAGTTGCTGATCTGGCGCGTGGAGCGGAAGGTCACGGGGTCTTGCATCATGTCTGGCAACGACCAGTAGACCTGCTCCGAGCCGCAGGTTACGTCAGGAGTGCAAAGCAGGGTCCATGAGCGGATTCCGGTCTGAGGGTCGCCGTAGTTGTATGCAAGGTACCAGCTGCGGTACTGGGTGAAGGTGCTGTCATTGCTTATTAGGTTGTAACTGCGTTTCTTGCTGTCCTCTGTCAGGGGTTTGATCACCGGTTTCTTCTGAGGCTTCTCGACCTCGGGTTGCGCAGCGGGGGCGGCAGCTTCGGCCGCTGCTTCGGCCTCAGCGCGCTTCTCCTCAGCCCGTGTGGCAAAGGTGTCGCCGCGAATGGCATGATCGTTCATGTCCTCCACCGGCTGCATTGCCGCGGCTGCCGCGTTGGAGTTCTCTTCCGCGCCGCTGCCACTGCTGTTGCTGCCGCCTGCGCCACCCCCGCCCTGTTCGGTGTCATCTTTCAAGCTCGCCTGGTAGGCGTCCACATCCAACAGTGCGGGAATGTTACCACCCTCCAGATCATCGTAGGTGATCCTAAAGCCCTCCTGGAAGGGTTGCCGCTTGCGGATGCCCAACAAGTTGCTCAGGCGGCTGTGGGTGAAGTCCACCCCGCATCCTGGCAGCAAAATGATGTCTGGATGGAAGGCTTCGTTTGTATATACCCCAGGCATGACAAGACCAGTGACGGGGTCAAACCCCAGTCTGAAGTTGCGGGTGTCAAACTTTACCCCGATGTCGCTTTCCAGAACCCCGTTCTGTCTGCCCACTTTCAAGTAGTGCTCCACGATCGCGTTGTTCATAAGGTCTATGGTCATGGTCTCGGAGTAGTTGCCCTCGGGCAGCGTGAACTCCACCCACTCGTATTTCAGCTCCACCTGATTGTCCTTAGTAGGCAAGCGCGACACCATCACCCGCGCCTTAAACTTATTGGTAAACATGAACTCGTTCACATTTGGCATGTTGGTATGCAGGATGGTTTTCAGGTCGCCGCCCCAGTGCGACCGGTCGTCAAGATTGATGGTCTGTGTGCTTCGAAGatctttctagaagatctcctacaatattctcagctgccatggaaaatcgatgttcttcttttattctctcaagattttcaggctgtatattaaaacttatattaagaactatgctaaccacctcatcaggaaccgttgtaggtggcgtgggttttcttggcaatcgactctcatgaaaactacgagctaaatattcaatatgttcctcttgaccaactttattctgcattttttttgaacgaggtttagagcaagcttcaggaaactgagacaggaattttattaaaaatttaaattttgaagaaagttcagggttaatagcatccattttttgctttgcaagttcctcagcattcttaacaaaagacgtctcttttgacatgtttaaagtttaaacctcctgtgtgaaattattatccgctcataattccacacattatacgagccggaagcataaagtgtaaagcctggggtgcctaatgagtgagctaactcacattaattgcgttgcgctcactgccaattgctttccagtcgggaaacctgtcgtgccagctgcattaatgaatcggccaacgcgcggggagaggcggtttgcgtattgggcgctcttccgcttcctcgctcactgactcgctgcgctcggtcgttcggctgcggcgagcggtatcagctcactcaaaggcggtaatacggttatccacagaatcaggggataacgcaggaaagaacatgtgagcaaaaggccagcaaaaggccaggaaccgtaaaaaggccgcgttgctggcgtttttccataggctccgcccccctgacgagcatcacaaaaatcgacgctcaagtcagaggtggcgaaacccgacaggactataaagataccaggcgtttccccctggaagctccctcgtgcgctctcctgttccgaccctgccgcttaccggatacctgtccgcctttctcccttcgggaagcgtggcgctttctcatagctcacgctgtaggtatctcagttcggtgtaggtcgttcgctccaagctgggctgtgtgcacgaaccccccgttcagcccgaccgctgcgccttatccggtaactatcgtcttgagtccaacccggtaagacacgacttatcgccactggcagcagccactggtaacaggattagcagagcgaggtatgtaggcggtgctacagagttcttgaagtggtggcctaactacggctacactagaaggacagtatttggtatctgcgctctgctgaagccagttaccttcggaaaaagagttggtagctcttgatccggcaaacaaaccaccgctggtagcggtggtttttttgtttgcaagcagcagattacgcgcagaaaaaaaggatctcaagaagatcctttgatcttttctacggggtctgacgctcagtggaacgaaaactcacgttaagggattttggtcatgagattatcaaaaaggatcttcacctagatccttttaaattaaaaatgaagttttaaatcaatctaaagtatatatgagtaaacttggtctgacagttaccaatgcttaatcagtgaggcacctatctcagcgatctgtctatttcgttcatccatagttgcctgactccccgtcgtgtagataactacgatacgggagggcttaccatctggccccagtgctgcaatgataccgcgagacccacgctcaccggctccagatttatcagcaataaaccagccagccggaagggccgagcgcagaagtggtcctgcaactttatccgcctccatccagtctattaattgttgccgggaagctagagtaagtagttcgccagttaatagtttgcgcaacgttgttgccattgctacaggcatcgtggtgtcacgctcgtcgtttggtatggcttcattcagctccggttcccaacgatcaaggcgagttacatgatcccccatgttgtgcaaaaaagcggttagctccttcggtcctccgatcgttgtcagaagtaagttggccgcagtgttatcactcatggttatggcagcactgcataattctcttactgtcatgccatccgtaagatgcttttctgtgactggtgagtactcaaccaagtcattctgagaatagtgtatgcggcgaccgagttgctcttgcccggcgtcaatacgggataataccgcgccacatagcagaactttaaaagtgctcatcattggaaaacgttcttcggggcgaaaactctcaaggatcttaccgctgttgagatccagttcgatgtaacccactcgtgcacccaactgatcttcagcatcttttactttcaccagcgtttctgggtgagcaaaaacaggaaggcaaaatgccgcaaaaaagggaataagggcgacacggaaatgttgaatactcatactcttcctttttcaatattattgaagcatttatcagggttattgtctcatgagcggatacatatttgaatgtatttagaaaaataaacaaataggggttccgcgcacatttccccgaaaagtgccacctgacgtctaagaaaccattattatcatgacattaacctataaaaataggcgtatcacgaggcc

**Block 5 (17998-25062)**

Plasmid name: pAd5-B5

Plasmid size: 10053 bp

Ad5 insert: 7065 bp

gcccctgcagccgaattatattatttttgccaaataatttttaacaaaagctctgaagtcttcttcatttaaattcttagatgatacttcatctggaaaattgtcccaattagtagcatcacgctgtgagtaagttctaaaccatttttttattgttgtattatctctaatcttactactcgatgagttttcggtattatctctatttttaacttggagcaggttccattcattgtttttttcatcatagtgaataaaatcaactgctttaacacttgtgcctgaacaccatatccatccggcgtaatacgactcactatagggagagcggccgccagatcttccggatggctcgagtttttcagcaagatcttcgaaGTAGAATGGAAGACATCAACTTTGCGTCTCTGGCCCCGCGACACGGCTCGCGCCCGTTCATGGGAAACTGGCAAGATATCGGCACCAGCAATATGAGCGGTGGCGCCTTCAGCTGGGGCTCGCTGTGGAGCGGCATTAAAAATTTCGGTTCCACCGTTAAGAACTATGGCAGCAAGGCCTGGAACAGCAGCACAGGCCAGATGCTGAGGGATAAGTTGAAAGAGCAAAATTTCCAACAAAAGGTGGTAGATGGCCTGGCCTCTGGCATTAGCGGGGTGGTGGACCTGGCCAACCAGGCAGTGCAAAATAAGATTAACAGTAAGCTTGATCCCCGCCCTCCCGTAGAGGAGCCTCCACCGGCCGTGGAGACAGTGTCTCCAGAGGGGCGTGGCGAAAAGCGTCCGCGCCCCGACAGGGAAGAAACTCTGGTGACGCAAATAGACGAGCCTCCCTCGTACGAGGAGGCACTAAAGCAAGGCCTGCCCACCACCCGTCCCATCGCGCCCATGGCTACCGGAGTGCTGGGCCAGCACACACCCGTAACGCTGGACCTGCCTCCCCCCGCCGACACCCAGCAGAAACCTGTGCTGCCAGGCCCGACCGCCGTTGTTGTAACCCGTCCTAGCCGCGCGTCCCTGCGCCGCGCCGCCAGCGGTCCGCGATCGTTGCGGCCCGTAGCCAGTGGCAACTGGCAAAGCACACTGAACAGCATCGTGGGTCTGGGGGTGCAATCCCTGAAGCGCCGACGATGCTTCTGAATAGCTAACGTGTCGTATGTGTGTCATGTATGCGTCCATGTCGCCGCCAGAGGAGCTGCTGAGCCGCCGCGCGCCCGCTTTCCAAGATGGCTACCCCTTCGATGATGCCGCAGTGGTCTTACATGCACATCTCGGGCCAGGACGCCTCGGAGTACCTGAGCCCCGGGCTGGTGCAGTTTGCCCGCGCCACCGAGACGTACTTCAGCCTGAATAACAAGTTTAGAAACCCCACGGTGGCGCCTACGCACGACGTGACCACAGACCGGTCCCAGCGTTTGACGCTGCGGTTCATCCCTGTGGACCGTGAGGATACTGCGTACTCGTACAAGGCGCGGTTCACCCTAGCTGTGGGTGATAACCGTGTGCTGGACATGGCTTCCACGTACTTTGACATCCGCGGCGTGCTGGACAGGGGCCCTACTTTTAAGCCCTACTCTGGCACTGCCTACAACGCCCTGGCTCCCAAGGGTGCCCCAAATCCTTGCGAATGGGATGAAGCTGCTACTGCTCTTGAAATAAACCTAGAAGAAGAGGACGATGACAACGAAGACGAAGTAGACGAGCAAGCTGAGCAGCAAAAAACTCACGTATTTGGGCAGGCGCCTTATTCTGGTATAAATATTACAAAGGAGGGTATTCAAATAGGTGTCGAAGGTCAAACACCTAAATATGCCGATAAAACATTTCAACCTGAACCTCAAATAGGAGAATCTCAGTGGTACGAAACTGAAATTAATCATGCAGCTGGGAGAGTCCTTAAAAAGACTACCCCAATGAAACCATGTTACGGTTCATATGCAAAACCCACAAATGAAAATGGAGGGCAAGGCATTCTTGTAAAGCAACAAAATGGAAAGCTAGAAAGTCAAGTGGAAATGCAATTTTTCTCAACTACTGAGGCGACCGCAGGCAATGGTGATAACTTGACTCCTAAAGTGGTATTGTACAGTGAAGATGTAGATATAGAAACCCCAGACACTCATATTTCTTACATGCCCACTATTAAGGAAGGTAACTCACGAGAACTAATGGGCCAACAATCTATGCCCAACAGGCCTAATTACATTGCTTTTAGGGACAATTTTATTGGTCTAATGTATTACAACAGCACGGGTAATATGGGTGTTCTGGCGGGCCAAGCATCGCAGTTGAATGCTGTTGTAGATTTGCAAGACAGAAACACAGAGCTTTCATACCAGCTTTTGCTTGATTCCATTGGTGATAGAACCAGGTACTTTTCTATGTGGAATCAGGCTGTTGACAGCTATGATCCAGATGTTAGAATTATTGAAAATCATGGAACTGAAGATGAACTTCCAAATTACTGCTTTCCACTGGGAGGTGTGATTAATACAGAGACTCTTACCAAGGTAAAACCTAAAACAGGTCAGGAAAATGGATGGGAAAAAGATGCTACAGAATTTTCAGATAAAAATGAAATAAGAGTTGGAAATAATTTTGCCATGGAAATCAATCTAAATGCCAACCTGTGGAGAAATTTCCTGTACTCCAACATAGCGCTGTATTTGCCCGACAAGCTAAAGTACAGTCCTTCCAACGTAAAAATTTCTGATAACCCAAACACCTACGACTACATGAACAAGCGAGTGGTGGCTCCCGGGcTAGTGGACTGCTACATTAACCTTGGAGCACGCTGGTCCCTTGACTATATGGACAACGTCAACCCATTTAACCACCACCGCAATGCTGGCCTGCGCTACCGCTCAATGTTGCTGGGCAATGGTCGCTATGTGCCCTTCCACATCCAGGTGCCTCAGAAGTTCTTTGCCATTAAAAACCTCCTTCTCCTGCCGGGCTCATACACCTACGAGTGGAACTTCAGGAAGGATGTTAACATGGTTCTGCAGAGCTCCCTAGGAAATGACCTAAGGGTTGACGGAGCCAGCATTAAGTTTGATAGCATTTGCCTTTACGCCACCTTCTTCCCCATGGCCCACAACACCGCCTCCACGCTTGAGGCCATGCTTAGAAACGACACCAACGACCAGTCCTTTAACGACTATCTCTCCGCCGCCAACATGCTCTACCCTATACCCGCCAACGCTACCAACGTGCCCATATCCATCCCCTCCCGCAACTGGGCGGCTTTCCGCGGCTGGGCCTTCACGCGCCTTAAGACTAAGGAAACCCCATCACTGGGCTCGGGCTACGACCCTTATTACACCTACTCTGGCTCTATACCCTACCTAGATGGAACCTTTTACCTCAACCACACCTTTAAGAAGGTGGCCATTACCTTTGACTCTTCTGTCAGCTGGCCTGGCAATGACCGCCTGCTTACCCCCAACGAGTTTGAAATTAAGCGCTCAGTTGACGGGGAGGGTTACAACGTTGCCCAGTGTAACATGACCAAAGACTGGTTCCTGGTACAAATGCTAGCTAACTAtAACATTGGCTACCAGGGCTTCTATATCCCAGAGAGCTACAAGGACCGCATGTACTCCTTCTTTAGAAACTTCCAGCCCATGAGCCGTCAGGTGGTGGATGATACTAAATACAAGGACTACCAACAGGTGGGCATCCTACACCAACACAACAACTCTGGATTTGTTGGCTACCTTGCCCCCACCATGCGCGAAGGACAGGCCTACCCTGCTAACTTCCCCTATCCGCTTATAGGCAAGACCGCAGTTGACAGCATTACCCAGAAAAAGTTTCTTTGCGATCGCACCCTTTGGCGCATCCCATTCTCCAGTAACTTTATGTCCATGGGCGCACTCACAGACCTGGGCCAAAACCTTCTCTACGCCAACTCCGCCCACGCGCTAGACATGACTTTTGAGGTGGATCCCATGGACGAGCCCACCCTTCTTTATGTTTTGTTTGAAGTCTTTGACGTGGTCCGTGTGCACCaGCCGCACCGCGGCGTCATCGAAACCGTGTACCTGCGCACGCCCTTCTCGGCCGGCAACGCCACAACATAAAGAAGCAAGCAACATCAACAACAGCTGCCGCCATGGGCTCCAGTGAGCAGGAACTGAAAGCCATTGTCAAAGATCTTGGTTGTGGGCCATATTTTTTGGGCACCTATGACAAGCGCTTTCCAGGCTTTGTTTCTCCACACAAGCTCGCCTGCGCCATAGTCAATACGGCCGGTCGCGAGACTGGGGGCGTACACTGGATGGCCTTTGCCTGGAACCCGCACTCAAAAACATGCTACCTCTTTGAGCCCTTTGGCTTTTCTGACCAGCGACTCAAGCAGGTTTACCAGTTTGAGTACGAGTCACTCCTGCGCCGTAGCGCCATTGCTTCTTCCCCCGACCGCTGTATAACGCTGGAAAAGTCCACCCAAAGCGTACAGGGGCCCAACTCGGCCGCCTGTGGACTATTCTGCTGCATGTTTCTCCACGCCTTTGCCAACTGGCCCCAAACTCCCATGGATCACAACCCCACCATGAACCTTATTACCGGGGTACCCAACTCCATGCTCAACAGTCCCCAGGTACAGCCCACCCTGCGTCGCAACCAGGAACAGCTCTACAGCTTCCTGGAGCGCCACTCGCCCTACTTCCGCAGCCACAGTGCGCAGATTAGGAGCGCCACTTCTTTTTGTCACTTGAAAAACATGTAAAAATAATGTACTAGAGACACTTTCAATAAAGGCAAATGCTTTTATTTGTACACTCTCGGGTGATTATTTACCCCCACCCTTGCCGTCTGCGCCGTTTAAAAATCAAAGGGGTTCTGCCGCGCATCGCTATGCGCCACTGGCAGGGACACGTTGCGATACTGGTGTTTAGTGCTCCACTTAAACTCAGGCACAACCATCCGCGGCAGCTCGGTGAAGTTTTCACTCCACAGGCTGCGCACCATCACCAACGCGTTTAGCAGGTCGGGCGCCGATATCTTGAAGTCGCAGTTGGGGCCTCCGCCCTGCGCGCGCGAGTTGCGATACACAGGGTTGCAGCACTGGAACACTATCAGCGCCGGGTGGTGCACGCTGGCCAGCACGCTCTTGTCGGAGATCAGATCCGCGTCCAGGTCCTCCGCGTTGCTCAGGGCGAACGGAGTCAACTTTGGTAGCTGCCTTCCCAAAAAGGGCGCGTGCCCAGGCTTTGAGTTGCACTCGCACCGTAGTGGCATCAAAAGGTGACCGTGCCCGGTCTGGGCGTTAGGATACAGCGCCTGCATAAAAGCCTTGATCTGCTTAAAAGCCACCTGAGCCTTTGCGCCTTCAGAGAAGAACATGCCGCAAGACTTGCCGGAAAACTGATTGGCCGGACAGGCCGCGTCGTGCACGCAGCACCTTGCGTCGGTGTTGGAGATCTGCACCACATTTCGGCCCCACCGGTTCTTCACGATCTTGGCCTTGCTAGACTGCTCCTTCAGCGCGCGCTGCCCGTTTTCGCTCGTCACATCCATTTCAATCACGTGCTCCTTATTTATCATAATGCTTCCGTGTAGACACTTAAGCTCGCCTTCGATCTCAGCGCAGCGGTGCAGCCACAACGCGCAGCCCGTGGGCTCGTGATGCTTGTAGGTCACCTCTGCAAACGACTGCAGGTACGCCTGCAGGAATCGCCCCATCATCGTCACAAAGGTCTTGTTGCTGGTGAAGGTCAGCTGCAACCCGCGGTGCTCCTCGTTCAGCCAGGTCTTGCATACGGCCGCCAGAGCTTCCACTTGGTCAGGCAGTAGTTTGAAGTTCGCCTTTAGATCGTTATCCACGTGGTACTTGTCCATCAGCGCGCGCGCAGCCTCCATGCCCTTCTCCCACGCAGACACGATCGGCACACTCAGCGGGTTCATCACCGTAATTTCACTTTCCGCTTCGCTGGGCTCTTCCTCTTCCTCTTGCGTCCGCATACCACGCGCCACTGGGTCGTCTTCATTCAGCCGCCGCACTGTGCGCTTACCTCCTTTGCCATGCTTGATTAGCACCGGTGGGTTGCTGAAACCCACCATTTGTAGCGCCACATCTTCTCTTTCTTCCTCGCTGTCCACGATTACCTCTGGTGATGGCGGGCGCTCGGGCTTGGGAGAAGGGCGCTTCTTTTTCTTCTTGGGCGCAATGGCCAAATCCGCCGCCGAGGTCGATGGCCGCGGGCTGGGTGTGCGCGGCACCAGCGCGTCTTGTGATGAGTCTTCCTCGTCCTCGGACTCGATACGCCGCCTCATCCGCTTTTTTGGGGGCGCCCGGGGAGGCGGCGGCGACGGGGACGGGGACGACACGTCCTCCATGGTTGGGGGACGTCGCGCCGCACCGCGTCCGCGCTCGGGGGTGGTTTCGCGCTGCTCCTCTTCCCGACTGGCCATTTCCTTCTCCTATAGGCAGAAAAAGATCATGGAGTCAGTCGAGAAGAAGGACAGCCTAACCGCCCCCTCTGAGTTCGCCACCACCGCCTCCACCGATGCCGCCAACGCGCCTACCACCTTCCCCGTCGAGGCACCCCCGCTTGAGGAGGAGGAAGTGATTATCGAGCAGGACCCAGGTTTTGTAAGCGAAGACGACGAGGACCGCTCAGTACCAACAGAGGATAAAAAGCAAGACCAGGACAACGCAGAGGCAAACGAGGAACAAGTCGGGCGGGGGGACGAAAGGCATGGCGACTACCTAGATGTGGGAGACGACGTGCTGTTGAAGCATCTGCAGCGCCAGTGCGCCATTATCTGCGACGCGTTGCAAGAGCGCAGCGATGTGCCCCTCGCCATAGCGGATGTCAGCCTTGCCTACGAACGCCACCTATTCTCACCGCGCGTACCCCCCAAACGCCAAGAAAACGGCACATGCGAGCCCAACCCGCGCCTCAACTTCTACCCCGTATTTGCCGTGCCAGAGGTGCTTGCCACCTATCACATCTTTTTCCAAAACTGCAAGATACCCCTATCCTGCCGTGCCAACCGCAGCCGAGCGGACAAGCAGCTGGCCTTGCGGCAGGGCGCTGTCATACCTGATATCGCCTCGCTCAACGAAGTGCCAAAAATCTTTGAGGGTCTTGGACGCGACGAGAAGCGCGCGGCAAACGCTCTGCAACAGGAAAACAGCGAAAATGAAAGTCACTCTGGAGTGTTGGTGGAACTCGAGGGTGACAACGCGCGCCTAGCCGTACTAAAACGCAGCATCGAGGTCACCCACTTTGCCTACCCGGCACTTAACCTACCCCCCAAGGTCATGAGCACAGTCATGAGTGAGCTGATCGTGCGCCGTGCGCAGCCCCTGGAGAGGGATGCAAATTTGCAAGAACAAACAGAGGAGGGCCTACCCGCAGTTGGCGACGAGCAGCTAGCGCGCTGGCTTCAAACGCGCGAGCCTGCCGACTTGGAGGAGCGACGCAAACTAATGATGTTCGAACatctttctagaagatctcctacaatattctcagctgccatggaaaatcgatgttcttcttttattctctcaagattttcaggctgtatattaaaacttatattaagaactatgctaaccacctcatcaggaaccgttgtaggtggcgtgggttttcttggcaatcgactctcatgaaaactacgagctaaatattcaatatgttcctcttgaccaactttattctgcattttttttgaacgaggtttagagcaagcttcaggaaactgagacaggaattttattaaaaatttaaattttgaagaaagttcagggttaatagcatccattttttgctttgcaagttcctcagcattcttaacaaaagacgtctcttttgacatgtttaaagtttaaacctcctgtgtgaaattattatccgctcataattccacacattatacgagccggaagcataaagtgtaaagcctggggtgcctaatgagtgagctaactcacattaattgcgttgcgctcactgccaattgctttccagtcgggaaacctgtcgtgccagctgcattaatgaatcggccaacgcgcggggagaggcggtttgcgtattgggcgctcttccgcttcctcgctcactgactcgctgcgctcggtcgttcggctgcggcgagcggtatcagctcactcaaaggcggtaatacggttatccacagaatcaggggataacgcaggaaagaacatgtgagcaaaaggccagcaaaaggccaggaaccgtaaaaaggccgcgttgctggcgtttttccataggctccgcccccctgacgagcatcacaaaaatcgacgctcaagtcagaggtggcgaaacccgacaggactataaagataccaggcgtttccccctggaagctccctcgtgcgctctcctgttccgaccctgccgcttaccggatacctgtccgcctttctcccttcgggaagcgtggcgctttctcatagctcacgctgtaggtatctcagttcggtgtaggtcgttcgctccaagctgggctgtgtgcacgaaccccccgttcagcccgaccgctgcgccttatccggtaactatcgtcttgagtccaacccggtaagacacgacttatcgccactggcagcagccactggtaacaggattagcagagcgaggtatgtaggcggtgctacagagttcttgaagtggtggcctaactacggctacactagaaggacagtatttggtatctgcgctctgctgaagccagttaccttcggaaaaagagttggtagctcttgatccggcaaacaaaccaccgctggtagcggtggtttttttgtttgcaagcagcagattacgcgcagaaaaaaaggatctcaagaagatcctttgatcttttctacggggtctgacgctcagtggaacgaaaactcacgttaagggattttggtcatgagattatcaaaaaggatcttcacctagatccttttaaattaaaaatgaagttttaaatcaatctaaagtatatatgagtaaacttggtctgacagttaccaatgcttaatcagtgaggcacctatctcagcgatctgtctatttcgttcatccatagttgcctgactccccgtcgtgtagataactacgatacgggagggcttaccatctggccccagtgctgcaatgataccgcgagacccacgctcaccggctccagatttatcagcaataaaccagccagccggaagggccgagcgcagaagtggtcctgcaactttatccgcctccatccagtctattaattgttgccgggaagctagagtaagtagttcgccagttaatagtttgcgcaacgttgttgccattgctacaggcatcgtggtgtcacgctcgtcgtttggtatggcttcattcagctccggttcccaacgatcaaggcgagttacatgatcccccatgttgtgcaaaaaagcggttagctccttcggtcctccgatcgttgtcagaagtaagttggccgcagtgttatcactcatggttatggcagcactgcataattctcttactgtcatgccatccgtaagatgcttttctgtgactggtgagtactcaaccaagtcattctgagaatagtgtatgcggcgaccgagttgctcttgcccggcgtcaatacgggataataccgcgccacatagcagaactttaaaagtgctcatcattggaaaacgttcttcggggcgaaaactctcaaggatcttaccgctgttgagatccagttcgatgtaacccactcgtgcacccaactgatcttcagcatcttttactttcaccagcgtttctgggtgagcaaaaacaggaaggcaaaatgccgcaaaaaagggaataagggcgacacggaaatgttgaatactcatactcttcctttttcaatattattgaagcatttatcagggttattgtctcatgagcggatacatatttgaatgtatttagaaaaataaacaaataggggttccgcgcacatttccccgaaaagtgccacctgacgtctaagaaaccattattatcatgacattaacctataaaaataggcgtatcacgaggcc

**Block 6 (25043-30907)**

Plasmid name: pAd5-B6

Plasmid size: 8853 bp

Ad5 insert: 5865 bp

gcccctgcagccgaattatattatttttgccaaataatttttaacaaaagctctgaagtcttcttcatttaaattcttagatgatacttcatctggaaaattgtcccaattagtagcatcacgctgtgagtaagttctaaaccatttttttattgttgtattatctctaatcttactactcgatgagttttcggtattatctctatttttaacttggagcaggttccattcattgtttttttcatcatagtgaataaaatcaactgctttaacacttgtgcctgaacaccatatccatccggcgtaatacgactcactatagggagagcggccgccagatcttccggatggctcgagtttttcagcaagatcttcgaaAGCGACGCAAACTAATGATGGCCGCAGTGCTCGTTACCGTGGAGCTTGAGTGCATGCAGCGGTTCTTTGCTGACCCGGAGATGCAGCGCAAGCTAGAGGAAACATTGCACTACACCTTTCGACAGGGCTACGTACGCCAGGCCTGCAAGATCTCCAACGTGGAGCTCTGCAACCTGGTCTCCTACCTTGGAATTTTGCACGAAAACCGCCTTGGGCAAAACGTGCTTCATTCCACGCTCAAGGGCGAGGCGCGCCGCGACTACGTCCGCGACTGCGTTTACTTATTTCTATGCTACACCTGGCAGACGGCCATGGGCGTTTGGCAGCAGTGCTTGGAGGAGTGCAACCTCAAGGAGCTGCAGAAACTGCTAAAGCAAAACTTGAAGGACCTATGGACGGCCTTCAACGAGCGCTCCGTGGCCGCGCACCTGGCGGACATCATTTTCCCCGAACGCCTGCTTAAAACCCTGCAACAGGGTCTGCCAGACTTCACCAGTCAAAGCATGTTGCAGAACTTTAGGAACTTTATCCTAGAGCGCTCAGGAATCTTGCCCGCCACCTGCTGTGCACTTCCTAGCGACTTTGTGCCCATTAAGTACCGCGAATGCCCTCCGCCGCTTTGGGGCCACTGCTACCTTCTGCAGCTAGCCAACTACCTTGCCTACCACTCTGACATAATGGAAGACGTGAGCGGTGACGGTCTACTGGAGTGTCACTGTCGCTGCAACCTATGCACCCCGCACCGCTCCCTGGTTTGCAATTCGCAGCTGCTTAACGAAAGTCAAATTATCGGTACCTTTGAGCTGCAGGGTCCCTCGCCTGACGAAAAGTCCGCGGCTCCGGGGTTGAAACTCACTCCGGGGCTGTGGACGTCGGCTTACCTTCGCAAATTTGTACCTGAGGACTACCACGCCCACGAGATTAGGTTCTACGAAGACCAATCCCGCCCGCCtAATGCGGAGCTTACCGCCTGCGTCATTACCCAGGGCCACATTCTTGGCCAATTGCAAGCCATCAACAAAGCCCGCCAAGAGTTTCTGCTACGAAAGGGACGGGGGGTTTACTTGGACCCCCAGTCCGGCGAGGAGCTCAACCCAATCCCCCCGCCGCCGCAGCCCTATCAGCAGCAGCCGCGGGCCCTTGCTTCCCAGGATGGCACCCAAAAAGAAGCTGCAGCTGCCGCCGCCACCCACGGACGAGGAGGAATACTGGGACAGTCAGGCAGAGGAGGTTTTGGACGAGGAGGAGGAGGACATGATGGAAGACTGGGAGAGCCTAGACGAGGAAGCTTCCGAGGTCGAAGAGGTGTCAGACGAAACACCGTCACCCTCGGTCGCATTCCCCTCGCCGGCGCCCCAGAAATCGGCAACCGGTTCCAGCATGGCTACAACCTCCGCTCCTCAGGCGCCGCCGGCACTGCCCGTTCGCCGACCCAACCGTAGATGGGACACCACTGGAACCAGGGCCGGTAAGTCCAAGCAGCCGCCGCCGTTAGCCCAAGAGCAACAACAGCGCCAAGGCTACCGCTCATGGCGCGGGCACAAGAACGCCATAGTTGCTTGCTTGCAAGACTGTGGGGGCAACATCTCCTTCGCCCGCCGCTTTCTTCTCTACCATCACGGCGTGGCCTTCCCCCGTAACATCCTGCATTACTACCGTCATCTCTACAGCCCATACTGCACCGGCGGCAGCGGCAGcaACAGCAGCGGCCACACAGAAGCAAAGGCGACCGGATAGCAAGACTCTGACAAAGCCCAAGAAATCCACAGCGGCGGCAGCAGCAGGAGGAGGAGCGCTGCGTCTGGCGCCCAACGAACCCGTATCGACCCGCGAGCTTAGAAACAGGATTTTTCCCACTCTGTATGCTATATTTCAACAGAGCAGGGGCCAAGAACAAGAGCTGAAAATAAAAAACAGGTCTCTGCGATCCCTCACCCGCAGCTGCCTGTATCACAAAAGCGAAGATCAGCTTCGGCGCACGCTGGAAGACGCGGAGGCTCTCTTCAGTAAATACTGCGCGCTGACTCTTAAGGACTAGTTTCGCGCCCTTTCTCAAATTTAAGCGCGAAAACTACGTCATCTCCAGCGGCCACACCCGGCGCCAGCACCTGTtGTCAGCGCCATTATGAGCAAGGAAATTCCCACGCCCTACATGTGGAGTTACCAGCCACAAATGGGACTTGCGGCTGGAGCTGCCCAAGACTACTCAACCCGAATAAACTACATGAGCGCGGGACCCCACATGATATCCCGGGTCAACGGAATaCGCGCCCACCGAAACCGAATTCTCcTGGAACAGGCGGCTATTACCACCACACCTCGTAATAACCTTAATCCCCGTAGTTGGCCCGCTGCCCTGGTGTACCAGGAAAGTCCCGCTCCCACCACTGTGGTACTTCCCAGAGACGCCCAGGCCGAAGTTCAGATGACTAACTCAGGGGCGCAGCTTGCGGGCGGCTTTCGTCACAGGGTGCGGTCGCCCGGGCAGGGTATAACTCACCTGACAATCAGAGGGCGAGGTATTCAGCTCAACGACGAGTCGGTGAGCTCCTCGCTTGGTCTCCGTCCGGACGGGACATTTCAGATCGGCGGCGCCGGCCGctCTTCATTCACGCCTCGTCAGGCAATCCTAACTCTGCAGACCTCGTCCTCTGAGCCGCGCTCTGGAGGCATTGGAACTCTGCAATTTATTGAGGAGTTTGTGCCATCGGTCTACTTTAACCCCTTCTCGGGACCTCCCGGCCACTATCCGGATCAATTTATTCCTAACTTTGACGCGGTAAAGGACTCGGCGGACGGCTACGACTGAATGTTAAGTGGAGAGGCAGAGCAACTGCGCCTGAAACACCTGGTCCACTGTCGCCGCCACAAGTGCTTTGCCCGCGACTCCGGTGAGTTTTGCTACTTTGAATTGCCCGAGGATCATATCGAGGGCCCGGCGCACGGCGTCCGGCTTACCGCCCAGGGAGAGCTTGCCCGTAGCCTGATTCGGGAGTTTACCCAGCGCCCCCTGCTAGTTGAGCGGGACAGGGGACCCTGTGTTCTCACTGTGATTTGCAACTGTCCTAACCcTGGATTACATCAAGATCTTTGTTGCCATCTCTGTGCTGAGTATAATAAATACAGAAATTAAAATATACTGGGGCTCCTATCGCCATCCTGTAAACGCCACCGTCTTCACCCGCCCAAGCAAACCAAGGCGAACCTTACCTGGTACTTTTAACATCTCTCCCTCTGTGATTTACAACAGTTTCAACCCAGACGGAGTGAGTCTACGAGAGAACCTCTCCGAGCTCAGCTACTCCATCAGAAAAAACACCACCCTCCTTACCTGCCGGGAACGTACGAGTGCGTCACCGGCCGCTGCACCACACCTACCGCCTGACCGTAAACCAGACTTTTTCCGGACAGACCTCAATAACTCTGTTTACCAGAACAGGAGGTGAGCTTAGAAAACCCTTAGGGTATTAGGCCAAAGGCGCAGCTACTGTGGGGTTTATGAACAATTCAAGCAACTCTACGGGCTATTCTAATTCAGGTTTCTCTAGAATCGGGGTTGGGGTTATTCTCTGTCTTGTGATTCTCTTTATTCTTATACTAACGCTTCTCTGCCTAAGGCTCGCCGCCTGCTGTGTGCACATTTGCATTTATTGTCAGCTTTTTAAACGCTGGGGTCGCCACCCAAGATGATTAGGTACATAATCCTAGGTTTACTCACCCTTGCGTCAGCCCACGGTACCACCCAAAAGGTGGATTTTAAGGAGCCAGCCTGTAATGTTACATTCGCAGCTGAAGCTAATGAGTGCACCACTCTTATAAAATGCACCACAGAACATGAAAAGCTGCTTATTCGCCACAAAAACAAAATTGGCAAGTATGCTGTTTATGCTATTTGGCAGCCAGGTGACACTACAGAGTATAATGTTACAGTTTTCCAGGGTAAAAGTCATAAAACTTTTATGTATACTTTTCCATTTTATGAAATGTGCGACATTACCATGTACATGAGCAAACAGTATAAGTTGTGGCCCCCACAAAATTGTGTGGAAAACACTGGCACTTTCTGCTGCACTGCTATGCTAATTACAGTGCTCGCTTTGGTCTGTACCCTACTCTATATTAAATACAAAAGCAGACGCAGCTTTATTGAGGAAAAGAAAATGCCTTAATTTACTAAGTTACAAAGCTAATGTCACCACTAACTGCTTTACTCGCTGCTTGCAAAACAAATTCAAAAAGTTAGCATTATAATTAGAATAGGATTTAAACCCCCCGGTCATTTCCTGCTCAATACCATTCCCCTGAACAATTGACTCTATGTGGGATATGCTCCAGCGCTACAACCTTGAAGTCAGGCTTCCTGGATGTCAGCATCTGACTTTGGCCAGCACCTGTCCCGCGGATTTGTTCCAGTCCAACTACAGCGACCCACCCTAACAGAGATGACCAACACAACCAACGCGGCCGCCGCTACCGGACTTACATCTACCACAAATACACCCCAAGTTTCTGCCTTTGTCAATAACTGGGATAACTTGGGCATGTGGTGGTTCTCCATAGCGCTTATGTTTGTATGCCTTATTATTATGTGGCTCATCTGCTGCCTAAAGCGCAAACGCGCCCGACCACCCATCTATAGTCCCATCATTGTGCTACACCCAAACAATGATGGAATCCATAGATTGGACGGACTGAAACACATGTTCTTTTCTCTTACAGTATGATTAAATGAGACATGATTCCTCGAGTTTTTATATTACTGACCCTTGTTGCGCTTTTTTGTGCGTGCTCCACATTGGCTGCGGTTTCTCACATCGAAGTAGACTGCATTCCAGCCTTCACAGTCTATTTGCTTTACGGATTTGTCACCCTCACGCTCATCTGCAGCCTCATCACTGTGGTCATCGCCTTTATCCAGTGCATTGACTGGGTCTGTGTGCGCTTTGCATATCTCAGACACCATCCCCAGTACAGGGACAGGACTATAGCTGAGCTTCTTAGAATTCTTTAATTATGAAATTTACTGTGACTTTTCTGCTGATTATTTGCACCCTATCTGCGTTTTGTTCCCCGACCTCCAAGCCTCAAAGACATATATCATGCAGATTCACTCGTATATGGAATATTCCAAGTTGCTACAATGAAAAAAGCGATCTTTCCGAAGCCTGGTTATATGCAATCATCTCTGTTATGGTGTTCTGCAGTACCATCTTAGCCCTAGCTATATATCCCTACCTTGACATTGGCTGGAAcgcAATAGATGCCATGAACCACCCAACTTTCCCCGCGCCCGCTATGCTTCCACTGCAACAAGTTGTTGCCGGCGGCTTTGTCCCAGCCAATCAGCCTCGCCCacCTTCTCCCACCCCCACTGAAATCAGCTACTTTAATCTAACAGGAGGAGATGACTGACACCCTAGATCTAGAAATGGACGGAATTATTACAGAGCAGCGCCTGCTAGAAAGACGCAGGGCAGCGGCCGAGCAACAGCGCATGAATCAAGAGCTCCAAGACATGGTTAACTTGCACCAGTGCAAAAGGGGTATCTTTTGTCTGGTAAAGCAGGCCAAAGTCACCTACGACAGTAATACCACCGGACACCGCCTTAGCTACAAGTTGCCAACCAAGCGTCAGAAATTGGTGGTCATGGTGGGAGAAAAGCCCATTACCATAACTCAGCACTCGGTAGAAACCGAAGGCTGCATTCACTCACCTTGTCAAGGACCTGAGGATCTCTGCACCCTTATTAAGACCCTGTGCGGTCTCAAAGATCTTATTCCCTTTAACTAATAAAAAAAAATAATAAAGCATCACTTACTTAAAATCAGTTAGCAAATTTCTGTCCAGTTTATTCAGCATTCGAACatctttctagaagatctcctacaatattctcagctgccatggaaaatcgatgttcttcttttattctctcaagattttcaggctgtatattaaaacttatattaagaactatgctaaccacctcatcaggaaccgttgtaggtggcgtgggttttcttggcaatcgactctcatgaaaactacgagctaaatattcaatatgttcctcttgaccaactttattctgcattttttttgaacgaggtttagagcaagcttcaggaaactgagacaggaattttattaaaaatttaaattttgaagaaagttcagggttaatagcatccattttttgctttgcaagttcctcagcattcttaacaaaagacgtctcttttgacatgtttaaagtttaaacctcctgtgtgaaattattatccgctcataattccacacattatacgagccggaagcataaagtgtaaagcctggggtgcctaatgagtgagctaactcacattaattgcgttgcgctcactgccaattgctttccagtcgggaaacctgtcgtgccagctgcattaatgaatcggccaacgcgcggggagaggcggtttgcgtattgggcgctcttccgcttcctcgctcactgactcgctgcgctcggtcgttcggctgcggcgagcggtatcagctcactcaaaggcggtaatacggttatccacagaatcaggggataacgcaggaaagaacatgtgagcaaaaggccagcaaaaggccaggaaccgtaaaaaggccgcgttgctggcgtttttccataggctccgcccccctgacgagcatcacaaaaatcgacgctcaagtcagaggtggcgaaacccgacaggactataaagataccaggcgtttccccctggaagctccctcgtgcgctctcctgttccgaccctgccgcttaccggatacctgtccgcctttctcccttcgggaagcgtggcgctttctcatagctcacgctgtaggtatctcagttcggtgtaggtcgttcgctccaagctgggctgtgtgcacgaaccccccgttcagcccgaccgctgcgccttatccggtaactatcgtcttgagtccaacccggtaagacacgacttatcgccactggcagcagccactggtaacaggattagcagagcgaggtatgtaggcggtgctacagagttcttgaagtggtggcctaactacggctacactagaaggacagtatttggtatctgcgctctgctgaagccagttaccttcggaaaaagagttggtagctcttgatccggcaaacaaaccaccgctggtagcggtggtttttttgtttgcaagcagcagattacgcgcagaaaaaaaggatctcaagaagatcctttgatcttttctacggggtctgacgctcagtggaacgaaaactcacgttaagggattttggtcatgagattatcaaaaaggatcttcacctagatccttttaaattaaaaatgaagttttaaatcaatctaaagtatatatgagtaaacttggtctgacagttaccaatgcttaatcagtgaggcacctatctcagcgatctgtctatttcgttcatccatagttgcctgactccccgtcgtgtagataactacgatacgggagggcttaccatctggccccagtgctgcaatgataccgcgagacccacgctcaccggctccagatttatcagcaataaaccagccagccggaagggccgagcgcagaagtggtcctgcaactttatccgcctccatccagtctattaattgttgccgggaagctagagtaagtagttcgccagttaatagtttgcgcaacgttgttgccattgctacaggcatcgtggtgtcacgctcgtcgtttggtatggcttcattcagctccggttcccaacgatcaaggcgagttacatgatcccccatgttgtgcaaaaaagcggttagctccttcggtcctccgatcgttgtcagaagtaagttggccgcagtgttatcactcatggttatggcagcactgcataattctcttactgtcatgccatccgtaagatgcttttctgtgactggtgagtactcaaccaagtcattctgagaatagtgtatgcggcgaccgagttgctcttgcccggcgtcaatacgggataataccgcgccacatagcagaactttaaaagtgctcatcattggaaaacgttcttcggggcgaaaactctcaaggatcttaccgctgttgagatccagttcgatgtaacccactcgtgcacccaactgatcttcagcatcttttactttcaccagcgtttctgggtgagcaaaaacaggaaggcaaaatgccgcaaaaaagggaataagggcgacacggaaatgttgaatactcatactcttcctttttcaatattattgaagcatttatcagggttattgtctcatgagcggatacatatttgaatgtatttagaaaaataaacaaataggggttccgcgcacatttccccgaaaagtgccacctgacgtctaagaaaccattattatcatgacattaacctataaaaataggcgtatcacgaggcc

**Block 7 (30888-35938)**

Plasmid name: pAd5-B7

Plasmid size: 8039 bp

Ad5 insert: 5050 bp in reverse orientation with respect to the Ad5 reference genome sequence.

gcccctgcagccgaattatattatttttgccaaataatttttaacaaaagctctgaagtcttcttcatttaaattcttagatgatacttcatctggaaaattgtcccaattagtagcatcacgctgtgagtaagttctaaaccatttttttattgttgtattatctctaatcttactactcgatgagttttcggtattatctctatttttaacttggagcaggttccattcattgtttttttcatcatagtgaataaaatcaactgctttaacacttgtgcctgaacaccatatccatccggcgtaatacgactcactatagggagagcggccgccagatcttccggatggctcgagtttttcagcaagatGTTCGAACATCATCAATAATATACCTTATTTTGGATTGAAGCCAATATGATAATGAGGGGGTGGAGTTTGTGACGTGGCGCGGGGCGTGGGAACGGGGCGGGTGACGTAGGTTTTAGGGCGGAGTAACTTGTATGTGTTGGGAATTGTAGTTTTCTTAAAATGGGAAGTgACGTAACGTGGGAAAACGGAAGTGACGATTTGAGGAAGTTGTGGGTTTTTTGGCTTTCGTTTCTGGGCGTAGGTTCGCGTGCGGTTTTCTGGGTGTTTTTTGTGGACTTTAACCGTTACGTCATTTTTTAGTCCTATATATACTCGCTCTGCACTTGGCCCTTTTTTACACTGTGACTGATTGAGCTGGTGCCGTGTCGAGTGGTGTTTTTTTAATAGGTTTTCTTTTTTACTGGTAAGGCTGACTGTTATGGCTGCCGCTGTGGAAGCGCTGTATGTTGTTCTGGAGCGGGAGGGTGCTATTTTGCCTAGGCAGGAGGGTTTTTCAGGTGTTTATGTGTTTTTCTCTCCTATTAATTTTGTTATACCTCCTATGGGGGCTGTAATGTTGTCTCTACGCCTGCGGGTATGTATTCCCCCGGGCTATTTCGGTCGCTTTTTAGCACTGACCGATGTGAATCAACCTGATGTGTTTACCGAGTCTTACATTATGACTCCGGACATGACCGAGGAGCTGTCGGTGGTGCTTTTTAATCACGGTGACCAGTTTTTTTACGGTCACGCCGGCATGGCCGTAGTCCGTCTTATGCTTATAAGGGTTGTTTTTCCTGTTGTAAGACAGGCTTCTAATGTTTAAATGTTTTTTTGTTATTTTATTTTGTGTTTATGCAGAAACCCGCAGACATGTTTGAGAGAAAAATGGTGTCTTTTTCTGTGGTGGTTCCGGAGCTTACCTGCCTTTATCTGCATGAGCATGACTACGATGTGCTTTCTTTTTTGCGCGAGGCTTTGCCTGATTTTTTGAGCAGCACCTTGCATTTTATATCGCCGCCCATGCAACAAGCTTACATCGGGGCTACGCTGGTTAGCATAGCTCCGAGTATGCGTGTCATAATCAGTGTGGGTTCTTTTGTCATGGTTCCTGGCGGGGAAGTGGCCGCGCTGGTCCGTGCAGACCTGCACGATTATGTTCAGCTGGCCCTGCGAAGGGACCTACGGGATCGCGGTATTTTTGTTAATGTTCCGCTTTTGAATCTTATACAGGTCTGTGAGGAACCTGAATTTTTGCAATCATGATTCGCTGCTTGAGGCTGAAGGTGGAGGGCGCTCTGGAGCAGATTTTTACAATGGCCGGACTTAATATTCGGGATTTGCTTAGAGATATATTGAGAAGGTGGCGAGATGAGAATTATTTGGGCATGGTTGAAGGTGCTGGAATGTTTATAGAGGAGATTCACCCTGAAGGGTTTAGCCTTTACGTCCACTTGGACGTGAGGGCCGTTTGCCTTTTGGAAGCCATTGTGCAACATCTTACAAATGCCATTATCTGTTCTTTGGCTGTAGAGTTTGACCACGCCACCGGAGGGGAGCGCGTTCACTTAATAGATCTTCATTTTGAGGTTTTGGATAATCTTTTGGAATAAAAAAAAAAaCATGGTTCTTCCAGCTCTTCCCGCTCCTCCCGTGTGTGACTCGCAGAACGAATGTGTAGGTTGGCTGGGTGTGGCTTATTCTGCGGTGGTGGATGTTATCAGGGCAGCGGCGCATGAAGGAGTTTACATAGAACCCGAAGCCAGGGGGCGCCTGGATGCTTTGAGAGAGTGGATATACTACAACTACTACACAGAGCGATCTAAGCGGCGAGACCGGAGACGCAGATCTGTTTGTCACGCCCGCACCTGGTTTTGCTTCAGGAAATATGACTACGTCCGGCGTTCCATTTGGCATGACACTACGACCAACACGATCTCGGTTGTCTCGGCGCACTCCGTACAGTAGGGATCGTCTACCTCCTTTTGAGACAGAAACCCGCGCTACCATACTGGAGGATCATCCGCTGCTGCCCGAATGTAACACTTTGACAATGCACAACGTGAGTTACGTGCGAGGTCTTCCCTGCAGTGTGGGATTTACGCTGATTCAGGAATGGGTTGTTCCCTGGGATATGGTTCTAACGCGGGAGGAGCTTGTAATCCTGAGGAAGTGTATGCACGTGTGCCTGTGTTGTGCCAACATTGATATCATGACGAGCATGATGATCCATGGTTACGAGTCCTGGGCTCTCCACTGTCATTGTTCCAGTCCCGGTTCCCTGCAGTGTATAGCCGGCGGGCAGGTTTTGGCCAGCTGGTTTAGGATGGTGGTGGATGGCGCCATGTTTAATCAGAGGTTTATATGGTACCGGGAGGTGGTGAATTACAACATGCCAAAAGAGGTAATGTTTATGTCCAGCGTGTTTATGAGGGGTCGCCACTTAATCTACCTGCGCTTGTGGTATGATGGCCACGTGGGTTCTGTGGTCCCCGCCATGAGCTTTGGATACAGCGCCTTGCACTGTGGGATTTTGAACAATATTGTGGTGCTGTGCTGCAGTTACTGTGCTGATTTAAGTGAGATCAGGGTGCGCTGCTGTGCCCGGAGGACAAGGCGCCTTATGCTGCGGGCGGTGCGAATCATCGCTGAGGAGACCACTGCCATGTTGTATTCCTGCAGGACGGAGCGGCGGCGGCAGCAGTTTATTCGCGCGCTGCTGCAGCACCACCGCCCTATCCTGATGCACGATTATGACTCTACCCCCATGTAGGCGTGGACTTCTCCTTCGCCGCCCGTTAAGCAACCGCAAGTTGGACAGCAGCCTGTGGCTCAGCAGCTGGACAGCGACATGAACTTAAGTGAGCTGCCCGGGGAGTTTATTAATATCACTGATGAGCGTTTGGCTCGACAGGAAACCGTGTGGAATATAACACCTAAGAATATGTCTGTTACCCATGATATGATGCTTTTTAAGGCCAGCCGGGGAGAAAGGACTGTGTACTCTGTGTGTTGGGAGGGAGGTGGCAGGTTGAATACTAGGGTTCTGTGAGTTTGATTAAGGTACGGTGATCTGTATAAGCTATGTGGTGGTGGGGCTATACTACTGAATGAAAAATGACTTGAAATTTTCTGCAATTGAAAAATAAACACGTTGAAACATAACACAAACGATTCTTTATTCTTGGGCAATGTATGAAAAAGTGTAAGAGGATGTGGCAAATATTTCATTAATGTAGTTGTGGCCAGACCAGTCCCATGAAAATGACATAGAGTATGCACTTGGAGTTGTGTCTCCTGTTTCCTGTGTACCGTTTAGTGTAATGGTTAGTGTTACAGGTTTAGTTTTGTCTCCGTTTAAGTAAACTTGACTGACAATGTTACTTTTGGCAGTTTTACCGTGAGATTTTGGATAAGCTGATAGGTTAGGCATAAATCCAACAGCGTTTGTATAGGCTGTGCCTTCAGTAAGATCTCCATTTCTAAAGTTCCAATATTCTGGGTCCAGGAAGGAATTGTTTAGTAGCACTCCATTTTCGTCAAATCTTATAATAAGATGAGCACTTTGAACTGTTCCAGATATTGGAGCCAAACTGCCTTTAACAGCCAAAACTGAAACTGTAGCAAGTATTTGACTGCCACATTTTGTTAAGACCAAAGTGAGTTTAGCATCTTTCTCTGCATTTAGTCTACAGTTAGGAGATGGAGCTGGTGTGGTCCACAAAGTTAGCTTATCATTATTTTTGTTTCCTACTGTAATGGCACCTGTGCTGTCAAAACTAAGGCCAGTTCCTAGTTTAGGAACCATAGCCTTGTTTGAATCAAATTCTAGGCCATGGCCAATTTTTGTTTTGAGGGGATTTGTGTTTGGTGCATTAGGTGAACCAAATTCAAGCCCATCTCCTGCATTAATGGCTATGGCTGTAGCGTCAAACATCAACCCCTTGGCAGTGCTTAGGTTAACCTCAAGCTTTTTGGAATTGTTTGAAGCTGTAAACAAGTAAAGGCCTTTGTTGTAGTTAATATCCAAGTTGTGGGCTGAGTTTATAAAAAGAGGGCCCTGTCCTAGTCTTAGATTTAGTTGGTTTTGAGCATCAAACGGATAACTAACATCAAGTATAAGGCGTCTGTTTTGAGAATCAATCCTTAGTCCTCCTGCTACATTAAGTTGCATATTGCCTTGTGAATCAAAACCCAAGGCTCCAGTAACTTTAGTTTGCAAGGAAGTATTATTAATAGTCACACCTGGACCAGTTGCTACGGTCAAAGTGTTTAGGTCGTCTGTTACATGCAAAGGAGCCCCGTACTTTAGTCCTAGTTTTCCATTTTGTGTATAAATGGGCTCTTTCAAGTCAATGCCCAAGCTACCAGTGGCAGTAGTTAGAGGGGGTGAGGCAGTGATAGTAAGGGTACTGCTATCGGTGGTGGTGAGGGGGCCTGATGTTTGCAGGGCTAGCTTTCCTTCTGACACTGTGAGGGGTCCTTGGGTGGCAATGCTAAGTTTGGAGTCGTGCACGGTTAGCGGGGCCTGTGATTGCATGGTGAGTGTGTTGCCCGCGACCATTAGAGGTGCGGCGGCAGCCACAGTTAGGGCTTCTGAGGTAACTGTGAGGGGTGCAGATATTTCCAGGTTTATGTTTGACTTGGTTTTTTTGAGAGGTGGGCTCACAGTGGTTACATTTTGGGAGGTAAGGTTGCCGGCCTCGTCCAGAGAGAGGCCGTTGCCCATTTTGAGCGCAAGCATGCCATTGGAGGTAACTAGAGGTTCGGATAGGCGCAAAGAGAGTACCCCAGGGGGACTCTCTTGAAACCCATTGGGGGATACAAAGGGAGGAGTAAGAAAAGGCACAGTTGGAGGACCGGTTTCCGTGTCATATGGATACACGGGGTTGAAGGTATCTTCAGACGGTCTTGCGCGCTTCATCTGCAACAACATGAAGATAGTGGGTGCGGATGGACAGGAACAGGAGGAAACTGACATTCCATTTAGATTGTGGAGAAAGTTTGCAGCCAGGAGGAAGCTGCAATACCAGAGCTGGGAGGAGGGCAAGGAGGTGCTGCTGAATAAACTGGACAGTTCGAAGatctttctagaagatctcctacaatattctcagctgccatggaaaatcgatgttcttcttttattctctcaagattttcaggctgtatattaaaacttatattaagaactatgctaaccacctcatcaggaaccgttgtaggtggcgtgggttttcttggcaatcgactctcatgaaaactacgagctaaatattcaatatgttcctcttgaccaactttattctgcattttttttgaacgaggtttagagcaagcttcaggaaactgagacaggaattttattaaaaatttaaattttgaagaaagttcagggttaatagcatccattttttgctttgcaagttcctcagcattcttaacaaaagacgtctcttttgacatgtttaaagtttaaacctcctgtgtgaaattattatccgctcataattccacacattatacgagccggaagcataaagtgtaaagcctggggtgcctaatgagtgagctaactcacattaattgcgttgcgctcactgccaattgctttccagtcgggaaacctgtcgtgccagctgcattaatgaatcggccaacgcgcggggagaggcggtttgcgtattgggcgctcttccgcttcctcgctcactgactcgctgcgctcggtcgttcggctgcggcgagcggtatcagctcactcaaaggcggtaatacggttatccacagaatcaggggataacgcaggaaagaacatgtgagcaaaaggccagcaaaaggccaggaaccgtaaaaaggccgcgttgctggcgtttttccataggctccgcccccctgacgagcatcacaaaaatcgacgctcaagtcagaggtggcgaaacccgacaggactataaagataccaggcgtttccccctggaagctccctcgtgcgctctcctgttccgaccctgccgcttaccggatacctgtccgcctttctcccttcgggaagcgtggcgctttctcatagctcacgctgtaggtatctcagttcggtgtaggtcgttcgctccaagctgggctgtgtgcacgaaccccccgttcagcccgaccgctgcgccttatccggtaactatcgtcttgagtccaacccggtaagacacgacttatcgccactggcagcagccactggtaacaggattagcagagcgaggtatgtaggcggtgctacagagttcttgaagtggtggcctaactacggctacactagaaggacagtatttggtatctgcgctctgctgaagccagttaccttcggaaaaagagttggtagctcttgatccggcaaacaaaccaccgctggtagcggtggtttttttgtttgcaagcagcagattacgcgcagaaaaaaaggatctcaagaagatcctttgatcttttctacggggtctgacgctcagtggaacgaaaactcacgttaagggattttggtcatgagattatcaaaaaggatcttcacctagatccttttaaattaaaaatgaagttttaaatcaatctaaagtatatatgagtaaacttggtctgacagttaccaatgcttaatcagtgaggcacctatctcagcgatctgtctatttcgttcatccatagttgcctgactccccgtcgtgtagataactacgatacgggagggcttaccatctggccccagtgctgcaatgataccgcgagacccacgctcaccggctccagatttatcagcaataaaccagccagccggaagggccgagcgcagaagtggtcctgcaactttatccgcctccatccagtctattaattgttgccgggaagctagagtaagtagttcgccagttaatagtttgcgcaacgttgttgccattgctacaggcatcgtggtgtcacgctcgtcgtttggtatggcttcattcagctccggttcccaacgatcaaggcgagttacatgatcccccatgttgtgcaaaaaagcggttagctccttcggtcctccgatcgttgtcagaagtaagttggccgcagtgttatcactcatggttatggcagcactgcataattctcttactgtcatgccatccgtaagatgcttttctgtgactggtgagtactcaaccaagtcattctgagaatagtgtatgcggcgaccgagttgctcttgcccggcgtcaatacgggataataccgcgccacatagcagaactttaaaagtgctcatcattggaaaacgttcttcggggcgaaaactctcaaggatcttaccgctgttgagatccagttcgatgtaacccactcgtgcacccaactgatcttcagcatcttttactttcaccagcgtttctgggtgagcaaaaacaggaaggcaaaatgccgcaaaaaagggaataagggcgacacggaaatgttgaatactcatactcttcctttttcaatattattgaagcatttatcagggttattgtctcatgagcggatacatatttgaatgtatttagaaaaataaacaaataggggttccgcgcacatttccccgaaaagtgccacctgacgtctaagaaaccattattatcatgacattaacctataaaaataggcgtatcacgaggcc

**Modified Block 1: deletion of E1, insertion of multiple cloning sites**

Plasmid name: pAd5-B1ΔE1MCS

Deletion: 481-3530

Plasmid size: 3742 bp

MCS sequences highlighted in yellow

gcccctgcagccgaattatattatttttgccaaataatttttaacaaaagctctgaagtcttcttcatttaaattcttagatgatacttcatctggaaaattgtcccaattagtagcatcacgctgtgagtaagttctaaaccatttttttattgttgtattatctctaatcttactactcgatgagttttcggtattatctctatttttaacttggagcaggttccattcattgtttttttcatcatagtgaataaaatcaactgctttaacacttgtgcctgaacaccatatccatccggcgtaatacgactcactatagggagagcggccgccagatcttccggatggctcgagtttttcagcaagat**cttcgaaC**ATCATCAATAATATACCTTATTTTGGATTGAAGCCAATATGATAATGAGGGGGTGGAGTTTGTGACGTGGCGCGGGGCGTGGGAACGGGGCGGGTGACGTAGTAGTGTGGCGGAAGTGTGATGTTGCAAGTGTGGCGGAACACATGTAAGCGACGGATGTGGCAAAAGTGACGTTTTTGGTGTGCGCCGGTGTACACAGGAAGTGACAATTTTCGCGCGGTTTTAGGCGGATGTTGTAGTAAATTTGGGCGTAACCGAGTAAGATTTGGCCATTTTCGCGGGAAAACTGAATAAGAGGAAGTGAAATCTGAATAATTTTGTGTTACTCATAGCGCGTAATATTTGTCTAGGGCCGCGGGGACTTTGACCGTTTACGTGGAGACTCGCCCAGGTGTTTTTCTCAGGTGTTTTCCGCGTTCCGGGTCAAAGTTGGCGTTTTATTATTATAGTCAGCTGACGTGTAGTGTATTTATACCCGGgaattcGGATCCaccggtGATATCttaattaaGTCGACactagtTGGCTTAAGGGTGGGAAAGAATATATAAGGTGGGGGTCTTATGTAGTTTTGTATCTGTTTTGCAGCAGCCGCCGCCGCCATGAGCACCAACTCGTTTGATGGAAGCATTGTGAGCTCATATTTGACAACGCGCATGCCCCCATGGGCCGGGGTGCGTCAGAATGTGATGGGCTCCAGCATTGATGGTCGCCCCGTCCTGCCCGCAAACTCTACTACCTTGACCTACGAGA**TTCGAAC**atctttctagaagatctcctacaatattctcagctgccatggaaaatcgatgttcttcttttattctctcaagattttcaggctgtatattaaaacttatattaagaactatgctaaccacctcatcaggaaccgttgtaggtggcgtgggttttcttggcaatcgactctcatgaaaactacgagctaaatattcaatatgttcctcttgaccaactttattctgcattttttttgaacgaggtttagagcaagcttcaggaaactgagacaggaattttattaaaaatttaaattttgaagaaagttcagggttaatagcatccattttttgctttgcaagttcctcagcattcttaacaaaagacgtctcttttgacatgtttaaagtttaaacctcctgtgtgaaattattatccgctcataattccacacattatacgagccggaagcataaagtgtaaagcctggggtgcctaatgagtgagctaactcacattaattgcgttgcgctcactgccaattgctttccagtcgggaaacctgtcgtgccagctgcattaatgaatcggccaacgcgcggggagaggcggtttgcgtattgggcgctcttccgcttcctcgctcactgactcgctgcgctcggtcgttcggctgcggcgagcggtatcagctcactcaaaggcggtaatacggttatccacagaatcaggggataacgcaggaaagaacatgtgagcaaaaggccagcaaaaggccaggaaccgtaaaaaggccgcgttgctggcgtttttccataggctccgcccccctgacgagcatcacaaaaatcgacgctcaagtcagaggtggcgaaacccgacaggactataaagataccaggcgtttccccctggaagctccctcgtgcgctctcctgttccgaccctgccgcttaccggatacctgtccgcctttctcccttcgggaagcgtggcgctttctcatagctcacgctgtaggtatctcagttcggtgtaggtcgttcgctccaagctgggctgtgtgcacgaaccccccgttcagcccgaccgctgcgccttatccggtaactatcgtcttgagtccaacccggtaagacacgacttatcgccactggcagcagccactggtaacaggattagcagagcgaggtatgtaggcggtgctacagagttcttgaagtggtggcctaactacggctacactagaaggacagtatttggtatctgcgctctgctgaagccagttaccttcggaaaaagagttggtagctcttgatccggcaaacaaaccaccgctggtagcggtggtttttttgtttgcaagcagcagattacgcgcagaaaaaaaggatctcaagaagatcctttgatcttttctacggggtctgacgctcagtggaacgaaaactcacgttaagggattttggtcatgagattatcaaaaaggatcttcacctagatccttttaaattaaaaatgaagttttaaatcaatctaaagtatatatgagtaaacttggtctgacagttaccaatgcttaatcagtgaggcacctatctcagcgatctgtctatttcgttcatccatagttgcctgactccccgtcgtgtagataactacgatacgggagggcttaccatctggccccagtgctgcaatgataccgcgagacccacgctcaccggctccagatttatcagcaataaaccagccagccggaagggccgagcgcagaagtggtcctgcaactttatccgcctccatccagtctattaattgttgccgggaagctagagtaagtagttcgccagttaatagtttgcgcaacgttgttgccattgctacaggcatcgtggtgtcacgctcgtcgtttggtatggcttcattcagctccggttcccaacgatcaaggcgagttacatgatcccccatgttgtgcaaaaaagcggttagctccttcggtcctccgatcgttgtcagaagtaagttggccgcagtgttatcactcatggttatggcagcactgcataattctcttactgtcatgccatccgtaagatgcttttctgtgactggtgagtactcaaccaagtcattctgagaatagtgtatgcggcgaccgagttgctcttgcccggcgtcaatacgggataataccgcgccacatagcagaactttaaaagtgctcatcattggaaaacgttcttcggggcgaaaactctcaaggatcttaccgctgttgagatccagttcgatgtaacccactcgtgcacccaactgatcttcagcatcttttactttcaccagcgtttctgggtgagcaaaaacaggaaggcaaaatgccgcaaaaaagggaataagggcgacacggaaatgttgaatactcatactcttcctttttcaatattattgaagcatttatcagggttattgtctcatgagcggatacatatttgaatgtatttagaaaaataaacaaataggggttccgcgcacatttccccgaaaagtgccacctgacgtctaagaaaccattattatcatgacattaacctataaaaataggcgtatcacgaggcc

**Modified Block 6: deletion of E3, insertion of BamHI site**

Plasmid name: pAd5-B6ΔE3

Deletion: 27859-30803

Plasmid size: 5908 bp

BamHI site highlighted in yellow

gcccctgcagccgaattatattatttttgccaaataatttttaacaaaagctctgaagtcttcttcatttaaattcttagatgatacttcatctggaaaattgtcccaattagtagcatcacgctgtgagtaagttctaaaccatttttttattgttgtattatctctaatcttactactcgatgagttttcggtattatctctatttttaacttggagcaggttccattcattgtttttttcatcatagtgaataaaatcaactgctttaacacttgtgcctgaacaccatatccatccggcgtaatacgactcactatagggagagcggccgccagatcttccggatggctcgagtttttcagcaagatcttcgaaAGCGACGCAAACTAATGATGGCCGCAGTGCTCGTTACCGTGGAGCTTGAGTGCATGCAGCGGTTCTTTGCTGACCCGGAGATGCAGCGCAAGCTAGAGGAAACATTGCACTACACCTTTCGACAGGGCTACGTACGCCAGGCCTGCAAGATCTCCAACGTGGAGCTCTGCAACCTGGTCTCCTACCTTGGAATTTTGCACGAAAACCGCCTTGGGCAAAACGTGCTTCATTCCACGCTCAAGGGCGAGGCGCGCCGCGACTACGTCCGCGACTGCGTTTACTTATTTCTATGCTACACCTGGCAGACGGCCATGGGCGTTTGGCAGCAGTGCTTGGAGGAGTGCAACCTCAAGGAGCTGCAGAAACTGCTAAAGCAAAACTTGAAGGACCTATGGACGGCCTTCAACGAGCGCTCCGTGGCCGCGCACCTGGCGGACATCATTTTCCCCGAACGCCTGCTTAAAACCCTGCAACAGGGTCTGCCAGACTTCACCAGTCAAAGCATGTTGCAGAACTTTAGGAACTTTATCCTAGAGCGCTCAGGAATCTTGCCCGCCACCTGCTGTGCACTTCCTAGCGACTTTGTGCCCATTAAGTACCGCGAATGCCCTCCGCCGCTTTGGGGCCACTGCTACCTTCTGCAGCTAGCCAACTACCTTGCCTACCACTCTGACATAATGGAAGACGTGAGCGGTGACGGTCTACTGGAGTGTCACTGTCGCTGCAACCTATGCACCCCGCACCGCTCCCTGGTTTGCAATTCGCAGCTGCTTAACGAAAGTCAAATTATCGGTACCTTTGAGCTGCAGGGTCCCTCGCCTGACGAAAAGTCCGCGGCTCCGGGGTTGAAACTCACTCCGGGGCTGTGGACGTCGGCTTACCTTCGCAAATTTGTACCTGAGGACTACCACGCCCACGAGATTAGGTTCTACGAAGACCAATCCCGCCCGCCAAATGCGGAGCTTACCGCCTGCGTCATTACCCAGGGCCACATTCTTGGCCAATTGCAAGCCATCAACAAAGCCCGCCAAGAGTTTCTGCTACGAAAGGGACGGGGGGTTTACTTGGACCCCCAGTCCGGCGAGGAGCTCAACCCAATCCCCCCGCCGCCGCAGCCCTATCAGCAGCAGCCGCGGGCCCTTGCTTCCCAGGATGGCACCCAAAAAGAAGCTGCAGCTGCCGCCGCCACCCACGGACGAGGAGGAATACTGGGACAGTCAGGCAGAGGAGGTTTTGGACGAGGAGGAGGAGGACATGATGGAAGACTGGGAGAGCCTAGACGAGGAAGCTTCCGAGGTCGAAGAGGTGTCAGACGAAACACCGTCACCCTCGGTCGCATTCCCCTCGCCGGCGCCCCAGAAATCGGCAACCGGTTCCAGCATGGCTACAACCTCCGCTCCTCAGGCGCCGCCGGCACTGCCCGTTCGCCGACCCAACCGTAGATGGGACACCACTGGAACCAGGGCCGGTAAGTCCAAGCAGCCGCCGCCGTTAGCCCAAGAGCAACAACAGCGCCAAGGCTACCGCTCATGGCGCGGGCACAAGAACGCCATAGTTGCTTGCTTGCAAGACTGTGGGGGCAACATCTCCTTCGCCCGCCGCTTTCTTCTCTACCATCACGGCGTGGCCTTCCCCCGTAACATCCTGCATTACTACCGTCATCTCTACAGCCCATACTGCACCGGCGGCAGCGGCAGctACAGCAGCGGCCACACAGAAGCAAAGGCGACCGGATAGCAAGACTCTGACAAAGCCCAAGAAATCCACAGCGGCGGCAGCAGCAGGAGGAGGAGCGCTGCGTCTGGCGCCCAACGAACCCGTATCGACCCGCGAGCTTAGAAACAGGATTTTTCCCACTCTGTATGCTATATTTCAACAGAGCAGGGGCCAAGAACAAGAGCTGAAAATAAAAAACAGGTCTCTGCGATCCCTCACCCGCAGCTGCCTGTATCACAAAAGCGAAGATCAGCTTCGGCGCACGCTGGAAGACGCGGAGGCTCTCTTCAGTAAATACTGCGCGCTGACTCTTAAGGACTAGTTTCGCGCCCTTTCTCAAATTTAAGCGCGAAAACTACGTCATCTCCAGCGGCCACACCCGGCGCCAGCACCTGTtGTCAGCGCCATTATGAGCAAGGAAATTCCCACGCCCTACATGTGGAGTTACCAGCCACAAATGGGACTTGCGGCTGGAGCTGCCCAAGACTACTCAACCCGAATAAACTACATGAGCGCGGGACCCCACATGATATCCCGGGTCAACGGAATaCGCGCCCACCGAAACCGAATTCTCcTGGAACAGGCGGCTATTACCACCACACCTCGTAATAACCTTAATCCCCGTAGTTGGCCCGCTGCCCTGGTGTACCAGGAAAGTCCCGCTCCCACCACTGTGGTACTTCCCAGAGACGCCCAGGCCGAAGTTCAGATGACTAACTCAGGGGCGCAGCTTGCGGGCGGCTTTCGTCACAGGGTGCGGTCGCCCGGGCAGGGTATAACTCACCTGACAATCAGAGGGCGAGGTATTCAGCTCAACGACGAGTCGGTGAGCTCCTCGCTTGGTCTCCGTCCGGACGGGACATTTCAGATCGGCGGCGCCGGCCGctCTTCATTCACGCCTCGTCAGGCAATCCTAACTCTGCAGACCTCGTCCTCTGAGCCGCGCTCTGGAGGCATTGGAACTCTGCAATTTATTGAGGAGTTTGTGCCATCGGTCTACTTTAACCCCTTCTCGGGACCTCCCGGCCACTATCCGGATCAATTTATTCCTAACTTTGACGCGGTAAAGGACTCGGCGGACGGCTACGACTGAAggatccGGTCTCAAAGATCTTATTCCCTTTAACTAATAAAAAAAAATAATAAAGCATCACTTACTTAAAATCAGTTAGCAAATTTCTGTCCAGTTTATTCAGCATTCGAACatctttctagaagatctcctacaatattctcagctgccatggaaaatcgatgttcttcttttattctctcaagattttcaggctgtatattaaaacttatattaagaactatgctaaccacctcatcaggaaccgttgtaggtggcgtgggttttcttggcaatcgactctcatgaaaactacgagctaaatattcaatatgttcctcttgaccaactttattctgcattttttttgaacgaggtttagagcaagcttcaggaaactgagacaggaattttattaaaaatttaaattttgaagaaagttcagggttaatagcatccattttttgctttgcaagttcctcagcattcttaacaaaagacgtctcttttgacatgtttaaagtttaaacctcctgtgtgaaattattatccgctcataattccacacattatacgagccggaagcataaagtgtaaagcctggggtgcctaatgagtgagctaactcacattaattgcgttgcgctcactgccaattgctttccagtcgggaaacctgtcgtgccagctgcattaatgaatcggccaacgcgcggggagaggcggtttgcgtattgggcgctcttccgcttcctcgctcactgactcgctgcgctcggtcgttcggctgcggcgagcggtatcagctcactcaaaggcggtaatacggttatccacagaatcaggggataacgcaggaaagaacatgtgagcaaaaggccagcaaaaggccaggaaccgtaaaaaggccgcgttgctggcgtttttccataggctccgcccccctgacgagcatcacaaaaatcgacgctcaagtcagaggtggcgaaacccgacaggactataaagataccaggcgtttccccctggaagctccctcgtgcgctctcctgttccgaccctgccgcttaccggatacctgtccgcctttctcccttcgggaagcgtggcgctttctcatagctcacgctgtaggtatctcagttcggtgtaggtcgttcgctccaagctgggctgtgtgcacgaaccccccgttcagcccgaccgctgcgccttatccggtaactatcgtcttgagtccaacccggtaagacacgacttatcgccactggcagcagccactggtaacaggattagcagagcgaggtatgtaggcggtgctacagagttcttgaagtggtggcctaactacggctacactagaaggacagtatttggtatctgcgctctgctgaagccagttaccttcggaaaaagagttggtagctcttgatccggcaaacaaaccaccgctggtagcggtggtttttttgtttgcaagcagcagattacgcgcagaaaaaaaggatctcaagaagatcctttgatcttttctacggggtctgacgctcagtggaacgaaaactcacgttaagggattttggtcatgagattatcaaaaaggatcttcacctagatccttttaaattaaaaatgaagttttaaatcaatctaaagtatatatgagtaaacttggtctgacagttaccaatgcttaatcagtgaggcacctatctcagcgatctgtctatttcgttcatccatagttgcctgactccccgtcgtgtagataactacgatacgggagggcttaccatctggccccagtgctgcaatgataccgcgagacccacgctcaccggctccagatttatcagcaataaaccagccagccggaagggccgagcgcagaagtggtcctgcaactttatccgcctccatccagtctattaattgttgccgggaagctagagtaagtagttcgccagttaatagtttgcgcaacgttgttgccattgctacaggcatcgtggtgtcacgctcgtcgtttggtatggcttcattcagctccggttcccaacgatcaaggcgagttacatgatcccccatgttgtgcaaaaaagcggttagctccttcggtcctccgatcgttgtcagaagtaagttggccgcagtgttatcactcatggttatggcagcactgcataattctcttactgtcatgccatccgtaagatgcttttctgtgactggtgagtactcaaccaagtcattctgagaatagtgtatgcggcgaccgagttgctcttgcccggcgtcaatacgggataataccgcgccacatagcagaactttaaaagtgctcatcattggaaaacgttcttcggggcgaaaactctcaaggatcttaccgctgttgagatccagttcgatgtaacccactcgtgcacccaactgatcttcagcatcttttactttcaccagcgtttctgggtgagcaaaaacaggaaggcaaaatgccgcaaaaaagggaataagggcgacacggaaatgttgaatactcatactcttcctttttcaatattattgaagcatttatcagggttattgtctcatgagcggatacatatttgaatgtatttagaaaaataaacaaataggggttccgcgcacatttccccgaaaagtgccacctgacgtctaagaaaccattattatcatgacattaacctataaaaataggcgtatcacgaggcc

**Modified Block 7: deletion of E4, insertion of SpeI site**

Plasmid name: pAd5-B7ΔE4

Deletion: 32874-35461

Plasmid size: 5456 bp

SpeI site highlighted in yellow

GcccctgcagccgaattatattatttttgccaaataatttttaacaaaagctctgaagtcttcttcatttaaattcttagatgatacttcatctggaaaattgtcccaattagtagcatcacgctgtgagtaagttctaaaccatttttttattgttgtattatctctaatcttactactcgatgagttttcggtattatctctatttttaacttggagcaggttccattcattgtttttttcatcatagtgaataaaatcaactgctttaacacttgtgcctgaacaccatatccatccggcgtaatacgactcactatagggagagcggccgccagatcttccggatggctcgagtttttcagcaagatGTTCGAACATCATCAATAATATACCTTATTTTGGATTGAAGCCAATATGATAATGAGGGGGTGGAGTTTGTGACGTGGCGCGGGGCGTGGGAACGGGGCGGGTGACGTAGGTTTTAGGGCGGAGTAACTTGTATGTGTTGGGAATTGTAGTTTTCTTAAAATGGGAAGTgACGTAACGTGGGAAAACGGAAGTGACGATTTGAGGAAGTTGTGGGTTTTTTGGCTTTCGTTTCTGGGCGTAGGTTCGCGTGCGGTTTTCTGGGTGTTTTTTGTGGACTTTAACCGTTACGTCATTTTTTAGTCCTATATATACTCGCTCTGCACTTGGCCCTTTTTTACACTGTGACTGATTGAGCTGGTGCCGTGTCGAGTGGTGTTTTTTTAATAGGTTTTCTTTTTTACTGGTAAGGCTGACTGTTATGGCTGCCGCTGTGGAAGCGCTGTATGTTGTTCTGGAGCGGGAGGGTGCTATTTTGCCTAGGCAactagtGTGGGGCTATACTACTGAATGAAAAATGACTTGAAATTTTCTGCAATTGAAAAATAAACACGTTGAAACATAACACAAACGATTCTTTATTCTTGGGCAATGTATGAAAAAGTGTAAGAGGATGTGGCAAATATTTCATTAATGTAGTTGTGGCCAGACCAGTCCCATGAAAATGACATAGAGTATGCACTTGGAGTTGTGTCTCCTGTTTCCTGTGTACCGTTTAGTGTAATGGTTAGTGTTACAGGTTTAGTTTTGTCTCCGTTTAAGTAAACTTGACTGACAATGTTACTTTTGGCAGTTTTACCGTGAGATTTTGGATAAGCTGATAGGTTAGGCATAAATCCAACAGCGTTTGTATAGGCTGTGCCTTCAGTAAGATCTCCATTTCTAAAGTTCCAATATTCTGGGTCCAGGAAGGAATTGTTTAGTAGCACTCCATTTTCGTCAAATCTTATAATAAGATGAGCACTTTGAACTGTTCCAGATATTGGAGCCAAACTGCCTTTAACAGCCAAAACTGAAACTGTAGCAAGTATTTGACTGCCACATTTTGTTAAGACCAAAGTGAGTTTAGCATCTTTCTCTGCATTTAGTCTACAGTTAGGAGATGGAGCTGGTGTGGTCCACAAAGTTAGCTTATCATTATTTTTGTTTCCTACTGTAATGGCACCTGTGCTGTCAAAACTAAGGCCAGTTCCTAGTTTAGGAACCATAGCCTTGTTTGAATCAAATTCTAGGCCATGGCCAATTTTTGTTTTGAGGGGATTTGTGTTTGGTGCATTAGGTGAACCAAATTCAAGCCCATCTCCTGCATTAATGGCTATGGCTGTAGCGTCAAACATCAACCCCTTGGCAGTGCTTAGGTTAACCTCAAGCTTTTTGGAATTGTTTGAAGCTGTAAACAAGTAAAGGCCTTTGTTGTAGTTAATATCCAAGTTGTGGGCTGAGTTTATAAAAAGAGGGCCCTGTCCTAGTCTTAGATTTAGTTGGTTTTGAGCATCAAACGGATAACTAACATCAAGTATAAGGCGTCTGTTTTGAGAATCAATCCTTAGTCCTCCTGCTACATTAAGTTGCATATTGCCTTGTGAATCAAAACCCAAGGCTCCAGTAACTTTAGTTTGCAAGGAAGTATTATTAATAGTCACACCTGGACCAGTTGCTACGGTCAAAGTGTTTAGGTCGTCTGTTACATGCAAAGGAGCCCCGTACTTTAGTCCTAGTTTTCCATTTTGTGTATAAATGGGCTCTTTCAAGTCAATGCCCAAGCTACCAGTGGCAGTAGTTAGAGGGGGTGAGGCAGTGATAGTAAGGGTACTGCTATCGGTGGTGGTGAGGGGGCCTGATGTTTGCAGGGCTAGCTTTCCTTCTGACACTGTGAGGGGTCCTTGGGTGGCAATGCTAAGTTTGGAGTCGTGCACGGTTAGCGGGGCCTGTGATTGCATGGTGAGTGTGTTGCCCGCGACCATTAGAGGTGCGGCGGCAGCCACAGTTAGGGCTTCTGAGGTAACTGTGAGGGGTGCAGATATTTCCAGGTTTATGTTTGACTTGGTTTTTTTGAGAGGTGGGCTCACAGTGGTTACATTTTGGGAGGTAAGGTTGCCGGCCTCGTCCAGAGAGAGGCCGTTGCCCATTTTGAGCGCAAGCATGCCATTGGAGGTAACTAGAGGTTCGGATAGGCGCAAAGAGAGTACCCCAGGGGGACTCTCTTGAAACCCATTGGGGGATACAAAGGGAGGAGTAAGAAAAGGCACAGTTGGAGGACCGGTTTCCGTGTCATATGGATACACGGGGTTGAAGGTATCTTCAGACGGTCTTGCGCGCTTCATCTGCAACAACATGAAGATAGTGGGTGCGGATGGACAGGAACAGGAGGAAACTGACATTCCATTTAGATTGTGGAGAAAGTTTGCAGCCAGGAGGAAGCTGCAATACCAGAGCTGGGAGGAGGGCAAGGAGGTGCTGCTGAATAAACTGGACAGTTCGAAGatctttctagaagatctcctacaatattctcagctgccatggaaaatcgatgttcttcttttattctctcaagattttcaggctgtatattaaaacttatattaagaactatgctaaccacctcatcaggaaccgttgtaggtggcgtgggttttcttggcaatcgactctcatgaaaactacgagctaaatattcaatatgttcctcttgaccaactttattctgcattttttttgaacgaggtttagagcaagcttcaggaaactgagacaggaattttattaaaaatttaaattttgaagaaagttcagggttaatagcatccattttttgctttgcaagttcctcagcattcttaacaaaagacgtctcttttgacatgtttaaagtttaaacctcctgtgtgaaattattatccgctcataattccacacattatacgagccggaagcataaagtgtaaagcctggggtgcctaatgagtgagctaactcacattaattgcgttgcgctcactgccaattgctttccagtcgggaaacctgtcgtgccagctgcattaatgaatcggccaacgcgcggggagaggcggtttgcgtattgggcgctcttccgcttcctcgctcactgactcgctgcgctcggtcgttcggctgcggcgagcggtatcagctcactcaaaggcggtaatacggttatccacagaatcaggggataacgcaggaaagaacatgtgagcaaaaggccagcaaaaggccaggaaccgtaaaaaggccgcgttgctggcgtttttccataggctccgcccccctgacgagcatcacaaaaatcgacgctcaagtcagaggtggcgaaacccgacaggactataaagataccaggcgtttccccctggaagctccctcgtgcgctctcctgttccgaccctgccgcttaccggatacctgtccgcctttctcccttcgggaagcgtggcgctttctcatagctcacgctgtaggtatctcagttcggtgtaggtcgttcgctccaagctgggctgtgtgcacgaaccccccgttcagcccgaccgctgcgccttatccggtaactatcgtcttgagtccaacccggtaagacacgacttatcgccactggcagcagccactggtaacaggattagcagagcgaggtatgtaggcggtgctacagagttcttgaagtggtggcctaactacggctacactagaaggacagtatttggtatctgcgctctgctgaagccagttaccttcggaaaaagagttggtagctcttgatccggcaaacaaaccaccgctggtagcggtggtttttttgtttgcaagcagcagattacgcgcagaaaaaaaggatctcaagaagatcctttgatcttttctacggggtctgacgctcagtggaacgaaaactcacgttaagggattttggtcatgagattatcaaaaaggatcttcacctagatccttttaaattaaaaatgaagttttaaatcaatctaaagtatatatgagtaaacttggtctgacagttaccaatgcttaatcagtgaggcacctatctcagcgatctgtctatttcgttcatccatagttgcctgactccccgtcgtgtagataactacgatacgggagggcttaccatctggccccagtgctgcaatgataccgcgagacccacgctcaccggctccagatttatcagcaataaaccagccagccggaagggccgagcgcagaagtggtcctgcaactttatccgcctccatccagtctattaattgttgccgggaagctagagtaagtagttcgccagttaatagtttgcgcaacgttgttgccattgctacaggcatcgtggtgtcacgctcgtcgtttggtatggcttcattcagctccggttcccaacgatcaaggcgagttacatgatcccccatgttgtgcaaaaaagcggttagctccttcggtcctccgatcgttgtcagaagtaagttggccgcagtgttatcactcatggttatggcagcactgcataattctcttactgtcatgccatccgtaagatgcttttctgtgactggtgagtactcaaccaagtcattctgagaatagtgtatgcggcgaccgagttgctcttgcccggcgtcaatacgggataataccgcgccacatagcagaactttaaaagtgctcatcattggaaaacgttcttcggggcgaaaactctcaaggatcttaccgctgttgagatccagttcgatgtaacccactcgtgcacccaactgatcttcagcatcttttactttcaccagcgtttctgggtgagcaaaaacaggaaggcaaaatgccgcaaaaaagggaataagggcgacacggaaatgttgaatactcatactcttcctttttcaatattattgaagcatttatcagggttattgtctcatgagcggatacatatttgaatgtatttagaaaaataaacaaataggggttccgcgcacatttccccgaaaagtgccacctgacgtctaagaaaccattattatcatgacattaacctataaaaataggcgtatcacgaggcc
