# Supplementary material for: Seamless assembly of recombinant adenoviral genomes from high-copy plasmids
Source: PLoS One. 2018 Jun 27;13(6):e0199563. doi: 10.1371/journal.pone.0199563 (PMC6021080; doi:10.1371/journal.pone.0199563)
Supplement: S2 Fig — (DOCX) [file pone.0199563.s008.docx]

**S2 Fig**

**Sequencing E4 mutant viral DNAs**

**E4ORF3 initiation codon ATG → ATA**

**
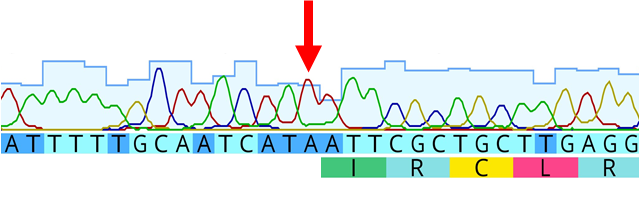
**

**E4ORF3 c-terminal FLAG tag**


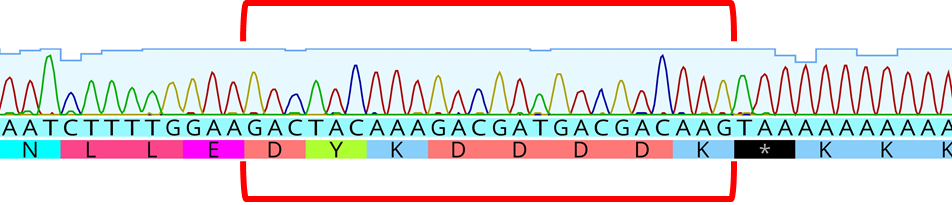


**Figure S2.** Amplified viral stocks were first treated with benzonase for 1 h at 37degC to eliminate unpackaged plasmid DNA. Viral DNAs were extracted by proteinase K digestion followed by purification over a column (GeneJet genomic DNA purification kit; Thermo Scientific). The relevant regions of the E4 locus were amplified with primers Ad-49 and Ad-50 (see Table S6). Column-purified PCR products (Monarch DNA clean up kit; NEB) were sequenced directly, with Ad5-49 as the sequencing primer.
